# The pleiotropic functions of Pri smORF peptides synchronize leg development regulators

**DOI:** 10.1371/journal.pgen.1011004

**Published:** 2023-10-30

**Authors:** Damien Markus, Aurore Pelletier, Muriel Boube, Fillip Port, Michael Boutros, François Payre, Benedikt Obermayer, Jennifer Zanet

**Affiliations:** 1 Molecular, Cellular and Developmental Biology Department (MCD), Centre de Biologie Intégrative (CBI), CNRS, UPS, University of Toulouse, Toulouse, France; 2 Division Signaling and Functional Genomics, German Cancer Research Center (DKFZ) and Heidelberg University, Heidelberg, Germany; 3 Core Unit Bioinformatics (CUBI), Berlin Institute of Health at Charité Universitätsmedizin-Berlin, Berlin, Germany; The University of North Carolina at Chapel Hill, UNITED STATES

## Abstract

The last decade witnesses the emergence of the abundant family of smORF peptides, encoded by small ORF (<100 codons), whose biological functions remain largely unexplored. Bioinformatic analyses here identify hundreds of putative smORF peptides expressed in *Drosophila* imaginal leg discs. Thanks to a functional screen in leg, we found smORF peptides involved in morphogenesis, including the pioneer smORF peptides Pri. Since we identified its target Ubr3 in the epidermis and *pri* was known to control leg development through poorly understood mechanisms, we investigated the role of Ubr3 in mediating *pri* function in leg. We found that *pri* plays several roles during leg development both in patterning and in cell survival. During larval stage, *pri* activates independently of Ubr3 tarsal transcriptional programs and Notch and EGFR signaling pathways, whereas at larval pupal transition, Pri peptides cooperate with Ubr3 to insure cell survival and leg morphogenesis. Our results highlight Ubr3 dependent and independent functions of Pri peptides and their pleiotropy. Moreover, we reveal that the smORF peptide family is a reservoir of overlooked developmental regulators, displaying distinct molecular functions and orchestrating leg development.

## Author summary

SmORF peptides are microproteins encoded by small ORFs, less than 100 codons, which have been ignored until recently due to their small size. Through a functional screen performed in *Drosophila* leg, we have revealed that the large family of smORF peptides contains novel regulators of morphogenesis. The pioneer smORF peptides Pri/Tal were known to be essential for leg development, but as their role remained poorly understood, we investigated whether its known target Ubr3, identified during epidermal differentiation, was necessary for leg development. We found that Pri peptides have distinct functions in this process. At the larval stage, independently of Ubr3, Pri peptides activate multiple actors, which together regulate the process of leg patterning. Then, at pupal stage, they induce in a Ubr3-dependent manner the activation of Svb transcription factor. The Pri/Ubr3/Svb module is required to protect cells from death during pupal stage and to preserve tissue integrity. Therefore, the pleiotropy of Pri peptides enables the activation of distinct targets over time required for the harmonious development of the legs.

## Introduction

The tremendous development of ribosome profiling, mass spectrometry and bioinformatics revealed the translation of thousands of small Open Reading Frames (smORF, <100 amino acids) in eukaryotes [1]. As they were considered non-coding due to their small size or their lack of homology, they have been overlooked until recently. SmORF peptides, also known as sORF peptides, microproteins, micropeptides or SEP (sORF encoded-peptides), are translated from smORF located in long non-coding (lnc) RNA, or previously alleged lncRNA, in intergenic region or in mRNA, in 5’, 3’UTR or within the coding sequence [2]. We are now facing thousands of smORF peptides that require functional characterization to distinguish bioactive smORF peptides from spurious ones. Interestingly, several studies focusing on the functions of particular smORF peptides have shown their role in the regulation of different cellular processes involved in development, metabolism or pathologies [1,3]. For instance, the smORF peptide encoded by Aw112010 lncRNA, highly expressed during infection, has been shown to be required for immunity response [4]. Also, Myoregulin and Dworf in mammals, and Sarcolamban in *Drosophila*, all translated from previously annotated lncRNA, control SERCA pump activity in muscles [5–7]. Since smORF peptides have been overlooked so far, they could constitute a reservoir of novel developmental regulators.

The *Drosophila* leg appears to be a good model for testing the biological role of genes encoding smORF peptides because, as an external and segmented organ, the morphology and the possible defects following genetic manipulation of these genes are easily observable in the adult leg. Fly leg development is stereotyped along a proximal-distal axis and relies on the coordination of cell patterning, cell growth, apoptosis and cell morphogenesis [8,9]. Indeed, during embryogenesis, presumptive organs named imaginal leg discs are formed. Then during larval stages, cells proliferate and a complex interplay between morphogens, signaling pathways and transcription factors subdivide the leg disc into different segments separated by folds, that prefigure the future joints. At pupal stage, the leg disc evaginates along the newly formed PD (proximo-distal) axis to form the adult leg composed of ten different segments articulated by joints [9].

In *Drosophila*, the pioneer smORF peptides Pri, encoded from a previously alleged lncRNA named *polished rice/tarsal-less* (*pri/tal*), have been firstly identified for their role both in leg formation, more specifically for the development of the tarsus [10], and in embryonic epidermal differentiation [11]. The *pri*/*tal* gene is polycistronic and encodes four Pri peptides, which exhibit a conserved motif among arthropods [10–12]. Several studies investigating Pri peptide functions during *Drosophila* lifespan have shown they are essential for development or maintenance of various tissues, such as embryonic epidermis and trachea, adult renal and intestinal stem cells and adult legs [10,11,13,14]. We have previously deciphered their molecular mode of action during epidermal differentiation and showed that Pri peptides interact with the E3 ubiquitin ligase Ubr3 to induce the specific recognition of the transcription factor Shavenbaby (Svb) and ubiquitination of its N-terminal domain. Svb undergoes a partial ubiquitin-dependent degradation of its N-terminal domain, switching it from a large transcriptional repressor (Svb^REP^) form to a shorter activator form (Svb^ACT^), enabling Svb to induce its target genes controlling epidermal differentiation [15,16].

We took advantage of the leg appendage to carry out a functional screen on putative smORF peptides identified specifically in this tissue at two developmental stages corresponding to different disc morphologies. Then, we found that depletion specifically in the leg of 23 of 93 genes encoding for smORF peptides with unknown functions resulted in defects in development. As the most differentially expressed gene at both developmental time points is *pri*/*tal* and their function is not well understood in the developing leg, we decided to investigate its role in the light of our findings [16]. Surprisingly, we found distinct functions for Pri during leg development. At the larval stage, Pri peptides are required for EGFR and Notch signaling pathways and transcriptional cascade activation, independently of Ubr3 and Svb. However, during pupal stage, the conserved Pri/Ubr3/Svb [17] module is involved to ensure cell survival, tarsi morphogenesis and tissue integrity. Thus, Pri peptides play pleiotropic functions within the same organ over time by controlling distinct actors, all of which together synchronize morphogenetic events ensuring harmonious leg development.

## Results

### smORF peptide family represents an overlooked reservoir of functional regulators during development

**Fig 1:**
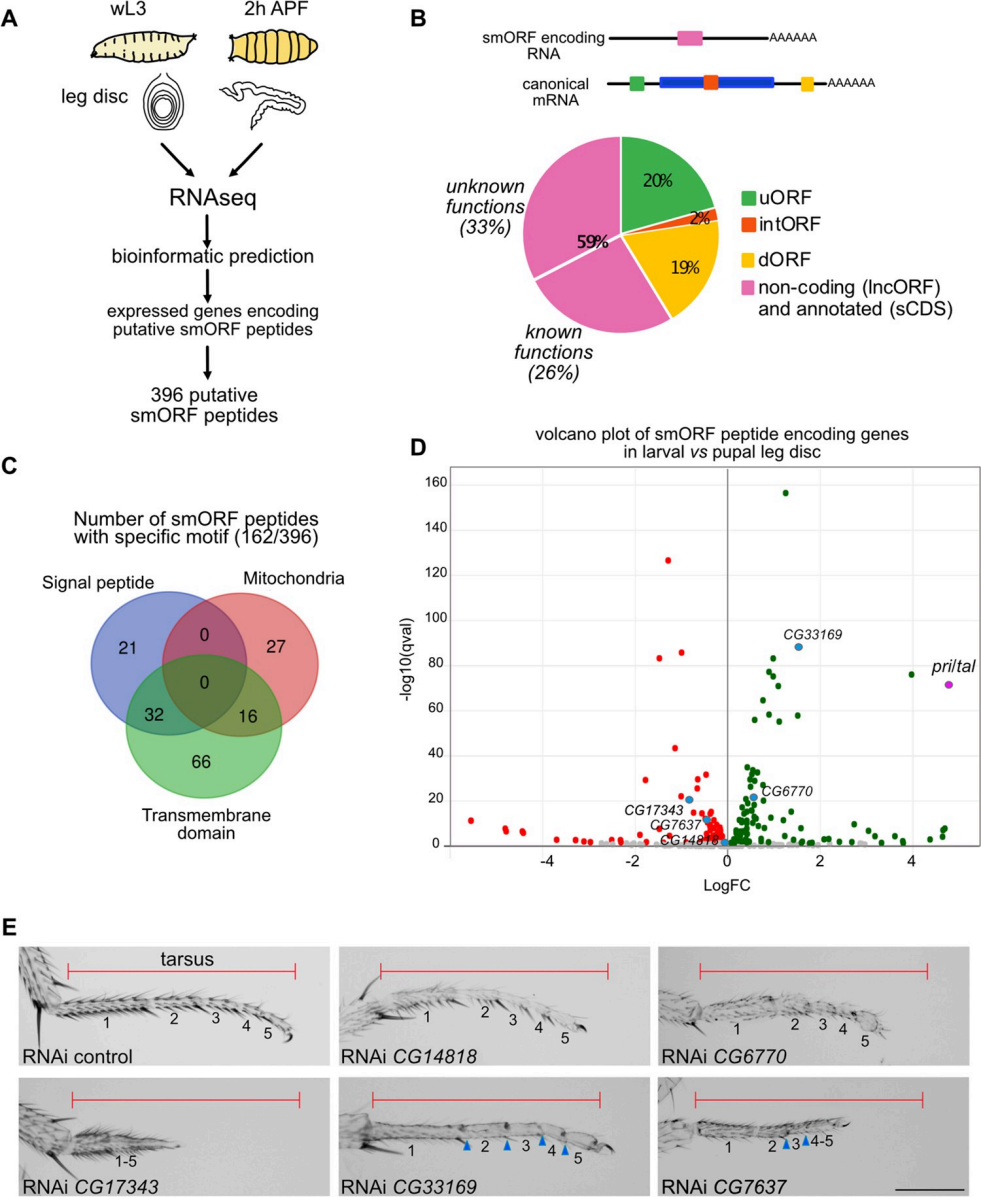
SmORF peptide family comprises a large number of developmental regulators. (A) Schematic representation of the pipeline to identify putative genes encoding smORF peptides expressed in the leg disc. RNAseq was done at two developmental time points of the leg disc, at wandering L3 (wL3) and 2 hours After Pupal Formation (APF). Then bioinformatic analysis was performed to predict smORF peptides from the genes expressed in the leg. (B) Diagram representing the different types of putative smORF peptides found in the leg discs both at wL3 and 2h APF. uORF stands for upstream ORF (green), intORF for internal ORF (orange), dORF for downstream ORF (yellow). SmORF located in monocistronic RNA (pink), which are annotated (sCDS for short coding sequence) or non-coding (lncORF), encoded for either characterized smORF peptides (known functions), or uncharacterized ones (unknown functions). (C) Venn diagram showing predicted protein motif (signal peptides, mitochondria targeting sequence, transmembrane domain) found in 162 putative smORF peptides over the 396 identified. (D) Volcano plot showing log2 fold change values (x-axis) by–log10 corrected p-values (y-axis) for genes encoding for putative smORF peptides between larval and pupal stages. Note that *pri*/*tal* gene is the most differentially expressed gene. (E) Examples of tarsal phenotypes obtained after loss of function of gene encoding smORF peptides. The UAS-RNAi is expressed under the control of *Dll*^EM212^-Gal4 at 29°C. Different defects can be observed, like shortening of the tarsus (compare length of the tarsus with the red line representing the length of the control), fusion of tarsal segments, incomplete joints (blue arrow-heads) and trichome defects. Scale bar = 200μm.

In order to find novel regulatory smORF peptides, we decided to identify candidates and to test their functionality by inducing their loss of function. We focused specifically on the *Drosophila* leg because, as an external organ, it facilitates phenotypic analyses and the identification of defects induced by loss of function of candidate genes. Furthermore, screening in the leg favors linking the type of defects to possible affected signaling pathways implicated for instance in proximo-distal axis patterning, tissue growth, joint formation or epidermal differentiation. To identify genes encoding putative smORF peptides, we performed differential expression analysis combined with a previously published smORF finding approach [18]. We thus generated transcriptomes of imaginal leg discs at two different stages of development, at wandering larval 3 stage (wL3) before pre-spiracle eversion, which indicates that the peak of ecdysone required for entry into metamorphosis has not yet occurred, and 2 hours APF (After Pupal Formation) (Fig 1A). Ecdysone signaling induces a transcriptional switch and leg evagination in the proximo-distal axis, possibly favoring our chances to find out regulatory smORF peptides. The bioinformatics analysis to identify smORFs is mainly based on the PhyloCSF method [19], which distinguishes coding and non-coding sequences based on substitution patterns in the whole genome alignment of 12 *Drosophila* species. This method allowed us to search for genes encoding for putative smORF peptides (Fig 1B) and to list 396 predicted ones (S1 Table), of which 103 are unannotated. Among them, prediction tools identify 162 smORF peptides with specific protein motifs, such as mitochondrial targeting sequence (MitoFates, DeepMito), peptide signal (SignalP 6.0) or transmembrane domain (TMHMM 2.0) (Fig 1C and S1 Table).

The majority of smORF (59%) are localized in a monocistronic RNA (216 genes), which are either annotated as coding (sCDS for short Coding Sequence) or non-coding (lncRNA and pseudogenes) [20]. Remaining smORF are found in canonical coding genes, localized in 5’UTR (uORF), 3’UTR (dORF) or within the main coding sequence (Fig 1B). Of the 216 putative smORF peptides, 96 have been characterized either through conservation among eukaryotes or through functional studies. Then, 120 putative smORF peptides remain with unknown function. Therefore, to figure out which of these smORF are functional and go beyond their theoretical identification, we induced their loss of function during leg development to test their role *in vivo*. Using both the available transgenic fly lines in the stock centers (Bloomington and VDRC) and newly generated fly lines, over these 120 genes, we were able to induce loss of function of 93 of them in the distal part of the leg using the Gal4/UAS system to drive either RNAi or gRNA (CRISPR/Cas9). We used the *Distal-less*-Gal4 driver (*Dll*^EM212^), which is expressed specifically in the leg from the distal tibia to the claws, the tarsus, during *Drosophila* development [21]. Over the 93 tested genes, the depletion of 23 of them impaired tarsus formation. We observed different types of tarsal defects, like fusion of segments, incomplete joints, necrosis, tarsi reduced size, altered cuticle formation or trichome pattern (Figs 1E and S1). The diversity of phenotypes suggests that smORF peptides are implicated in different cellular processes. Notably, loss of function of a high proportion of tested genes (24%), which encode putative smORF peptides, induces phenotypes. Therefore, our functional screen highlights the smORF peptides as a reservoir of novel developmental and cellular regulators.

Interestingly, differential expression analysis at two developmental time points shows a remarkable switch in gene expression between larval and pupal stage (946 genes with log2FC>1 and 827 with log2FC<1). Among the genes encoding putative smORF peptides, the most differentially expressed gene is the *pri*/*tal* gene (log2FC = 4,77; Fig 1D and S1 Table). Pri peptides have been discovered for their role in tarsus formation [10], where they are known to control tarsal patterning and joint formation [22–24]. Pri peptides are required for the establishment of the transcriptional program controlling tarsal segmentation, but the underlying mechanisms are not known. Also, it has been proposed that Pri peptides control joint morphogenesis through Svb and Notch regulation during pupal stage [24]. As the molecular mechanisms of action of Pri peptides remain not well understood during larval and pupal leg development, we then investigated their functions in the light of our recent findings and analyzed the role of Ubr3 in this process [16].

### Pri peptides play distinct roles at larval and pupal stages

**Fig 2:**
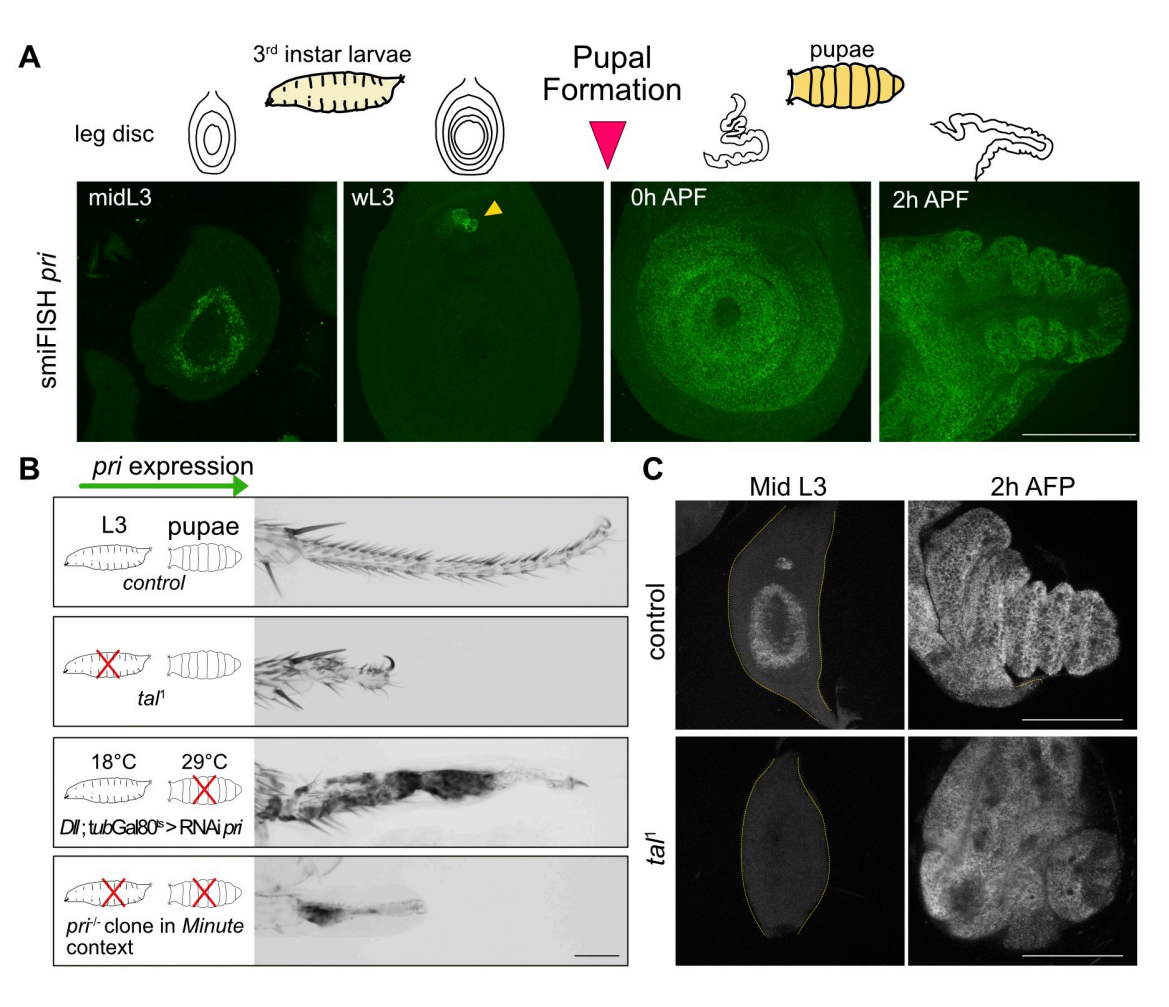
Pri peptides have distinct roles during leg development. (A) Fluorescent *in situ* hybridization of *pri* mRNA at different stages of leg disc development, schematized above by the drawings: from left to right, midL3 for mid third instar larvae 3, wL3 for wandering third instar larvae 3, 0h APF (After Pupal Formation) corresponds to the start of metamorphosis, 2h APF. *pri* expression is transient and pulsatile in a typical ring-shaped pattern in the presumptive tarsal region in the midL3 disc and *pri* expression is abolished in the wL3 disc (except *pri* expression observed in the chordotonal organ, yellow arrow-head). At the metamorphosis, corresponding to 0h APF, *pri* is strongly expressed in the whole distal region. In 2h APF disc, *pri* is expressed in the epithelium of the disc and in the peripodial membrane. (B) Phenotypes of adult leg obtained after stage specific *pri* depletion, schematized by a red cross, during larval or/and pupal stages. *pri* depletion during larval stage, here obtained by using the *tal*^*1*^ mutant, induces a strong reduction in tarsal size, fusion of the different tarsal segments and absence of tarsal joints. Loss of function specifically during pupal stage is induced by expressing UAS-RNAi *pri* under the control of *Dll*^EM212^-Gal4 driver and *tub*-Gal80^ts^ at 29°C, and leads to tissue integrity loss. To deplete *pri* during larval and pupal stages, *pri*^-/-^ (*tal*^S18^) clones in the *Minute* cellular context were induced specifically in the tarsus (*Dll*^EM212^>Flippase). The resulting phenotype cumulates the defects described above, up to the tarsus disappearance. (C) *pri in situ* fluorescent hybridization (smiFISH) in control and in *tal*^*1*^ leg discs. *pri* expression is absent in the *tal*^*1*^ mutant specifically during larval stage (midL3, outlined by the yellow dashed-line) and reactivated at metamorphosis (2h APF). Note the persistence of fusion of the tarsal segments in *tal*^*1*^ disc at 2h APF. All scale bars = 100μm.

As we observed a strong increase in *pri* expression between wL3 stage and 2h APF stages (Fig 1D), we analyzed *pri* mRNA localization by quantitative fluorescent *in situ* hybridization (smiFISH) [25]. As previously described [10], we observed *pri* mRNA at midL3 stage in the form of a ring-shaped pattern marking the presumptive territory of the tarsus, which stops at wL3 stage (except in the chordotonal organ) (Fig 2A). Then, at the onset of metamorphosis, *pri* is strongly reactivated in the whole leg disc and in the peripodial membrane (Fig 2A). The dynamic pattern of *pri* expression during leg development suggests different functions. To test this hypothesis, *pri* expression was specifically depleted in the tarsus at larval or/and pupal stages by using different genetic approaches (Fig 2B). To analyze the effect of *pri* depletion only during the larval stage, we used the *tal*^1^ mutant [10], in which *pri* expression is specifically absent during the larval stage in the leg, but unaffected at the onset of metamorphosis (Fig 2C). Indeed, in our hands, depletion of *pri* with RNAi during the larval stage was not efficient enough to get rid of the larval function of *pri*. *tal*^1^ allele affects the *cis*-regulatory genomic region controlling larval *pri* expression in the leg, named *pri*I [26]. Indeed, ectopic expression of *pri* under the control of *pri*I in *tal*^1^ mutant background restores tarsus morphology (S2A Fig). Thus, the absence of *pri* specifically during the larval stage leads to the fusion of the tarsal segments and then to a shorter tarsus (Fig 2B). To specifically delete *pri* at pupal stage, we used the Gal4/UAS and the thermo-inducible systems (*Dll*>RNAi *pri*; *tub-*Gal80^ts^). The absence of *pri* during pupal stage induces the loss of tissue integrity of the distal part of the leg (Fig 2B). Then, to perform *pri* depletion during both larval and pupal stages, we used the null allele mutant *pri*^S18^ [10] to induce large *pri*^-/-^ clones in *Minute* context specifically in the tarsus, using the *FRT*/*FLP* system, in which the Flippase is expressed under the control of *Dll*. Continuous depletion of *pri* over larval and pupal stages accumulates both phenotypes and results in a shorter and dramatically altered tarsus (Fig 2B). Therefore, during leg development, Pri smORF peptides exhibit distinct functions as they are required for proximo-distal axis patterning in the leg disc and consequently tarsal segmentation, and then at pupal stage, they are essential to ensure tissue integrity.

### Pri peptides are required for early steps of tarsal patterning

**Fig 3:**
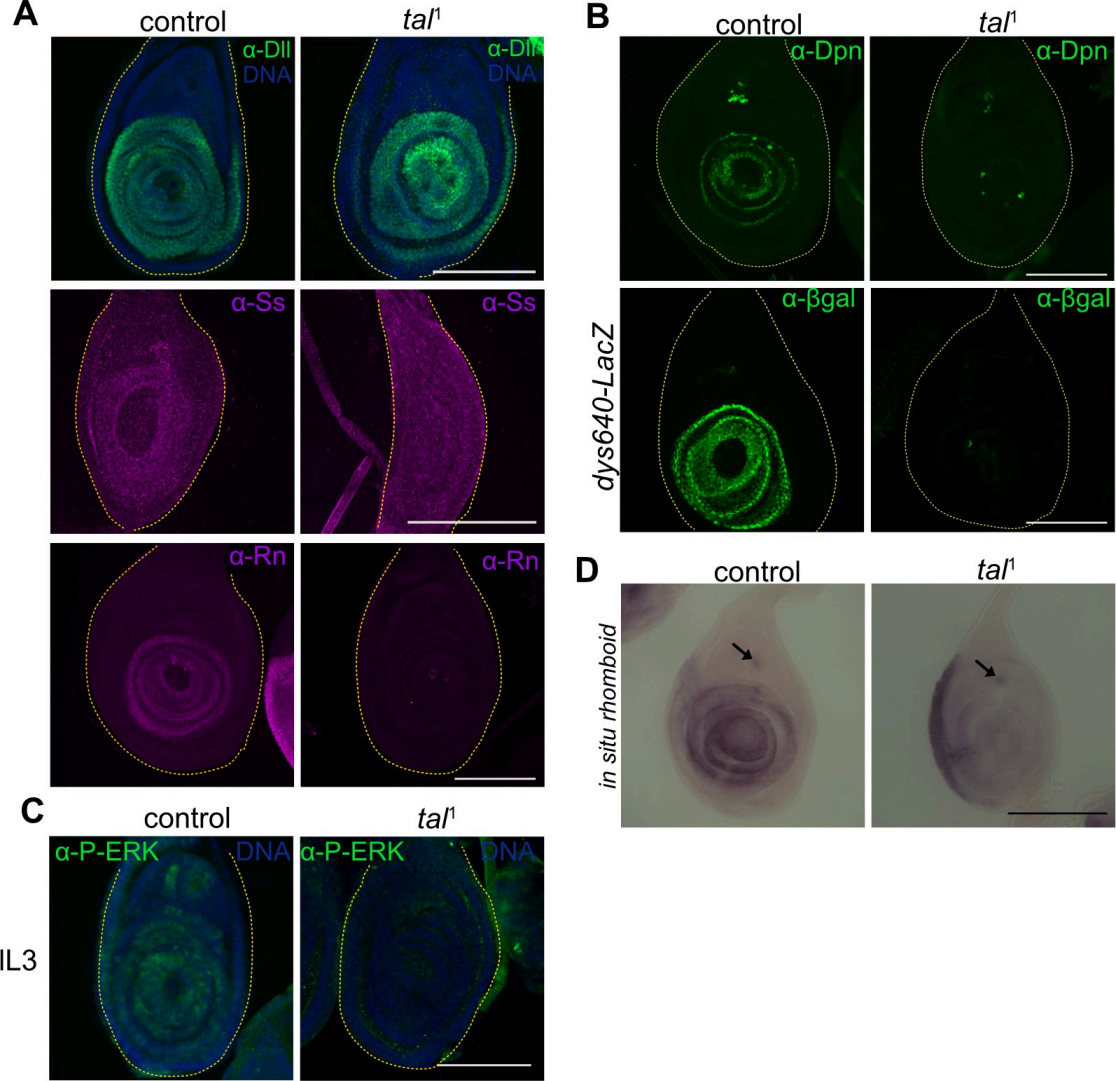
Pri peptides control tarsal patterning by activating transcriptional program and Notch and EGFR signaling pathways. (A) Immunostaining of Dll, Ss and Rn transcription factors in control and in *tal*^1^ mutant discs at L3 stage. Dll is present in both control and *tal*^1^ distal region, whereas Ss and Rn proteins are absent in the *tal*^*1*^ mutant leg discs. (B) Immunostainings of Deadpan (Dpn) and βGalactosidase (βGal) in a control and *tal*^*1*^ in L3 leg disc. *dys640*-LacZ is an enhancer of *dysfusion* gene directly activated by Notch, showing the absence of Notch signaling in the tarsal region. (C) The anti-P-ERK signal, used as a read-out of EGFR signaling, stains at late L3 stage (lL3) the whole tarsal region in the control while being absent in the *tal*^*1*^ mutant. The leg discs are outlined by the yellow dashed-line. *In situ* hybridization of *rhomboid* mRNA in control and in *tal*^1^ leg discs. (D) The *rhomboid* mRNA is observed in the presumptive tarsal region in concentric ring pattern. In the *tal*^*1*^ mutant, *rhomboid* expression is abrogated in the disc except in the chordotonal organ (black arrow). All scale bars = 100μm.

During larval stage, tarsal patterning is regulated by multiple actors, that define each segment composing the future tarsus. During the first and the second instar larvae, the morphogens Hedgehog (Hh), Decapentaplegic (Dpp) and Wingless (Wg) establish the anterior-posterior and dorso-ventral axis in the leg disc [27]. Consequently, the expression of *Dll* is activated in the leg disc, then defining the tarsal region during L3 stage [28]. Also, during early L3 stage, the EGFR signaling pathway is activated through the integration of the signal of Wg, Dpp and Dll at the center of the disc, known as the EGFR organizing center (EOC), and will govern the identity of the pretarsus. At mid-late L3 stage, a second wave of EGFR signaling (non-EOC), mostly dependent on the metalloprotease Rhomboid and the ligand Spitz, is activated in the tarsal region [29–31]. Thus, both EGFR and Dll subdivide the medial tarsal region and allow the expression of *spineless* (*ss*), which in turn induces *rotund* (*rn*), both TFs being necessary for subsequent tarsal patterning [22,32]. Furthermore, Notch signaling is required for patterning boundaries between segments, which prefigure joint formation [33]. Notably, *rn* is necessary for Notch pathway activation [34] (S2D Fig). During tarsal patterning, it was previously shown in *tal*^1^ and *tal*^KG^ mutants, or in *tal*^S18^ mutant clones, that *pri* was required for activating *ss* and *rn* transcription [10,22,23]. Here we showed in the *tal*^1^ mutant, in which *pri* expression is specifically abrogated in leg disc at larval stage, that Dll protein is still present while Ss and Rn are absent (Figs 3A and S2B). We then investigated at which stage of the regulatory cascade *pri* was acting. We observed that Notch signaling pathway, which is activated from the larval stage, was absent in the presumptive region of the tarsus in *tal*^1^ (Fig 3B), as confirmed by the absence of Deadpan (Dpn) protein and *dysfusion*-lacZ reporter line activity, both direct targets of the Notch pathway [35]. As EGFR is important for limiting Notch signaling at joint boundaries [36], we stained *tal*^1^ L3 leg discs with anti-Phospho-ERK antibody, a marker of MAPK activity used as a read-out of active EGFR signaling pathway, and revealed its absence in the tarsal region (Fig 3C). Furthermore, we found that in *tal*^1^ *rhomboid* mRNA was absent in tarsal region, showing that the second wave of EGFR activation is compromised (Fig 3D). Nevertheless, in the absence of *pri*, the initial EGFR wave is activated as indicated by the presence of the TF Clawless specific from the pretarsus and the formation of the claws (S2C Fig) [37]. Therefore, our data reveals that Pri peptides are required for *rhomboid* transcription, and consequently EGFR signaling, activation of the tarsal transcriptional program and Notch signaling.

**Fig 4:**
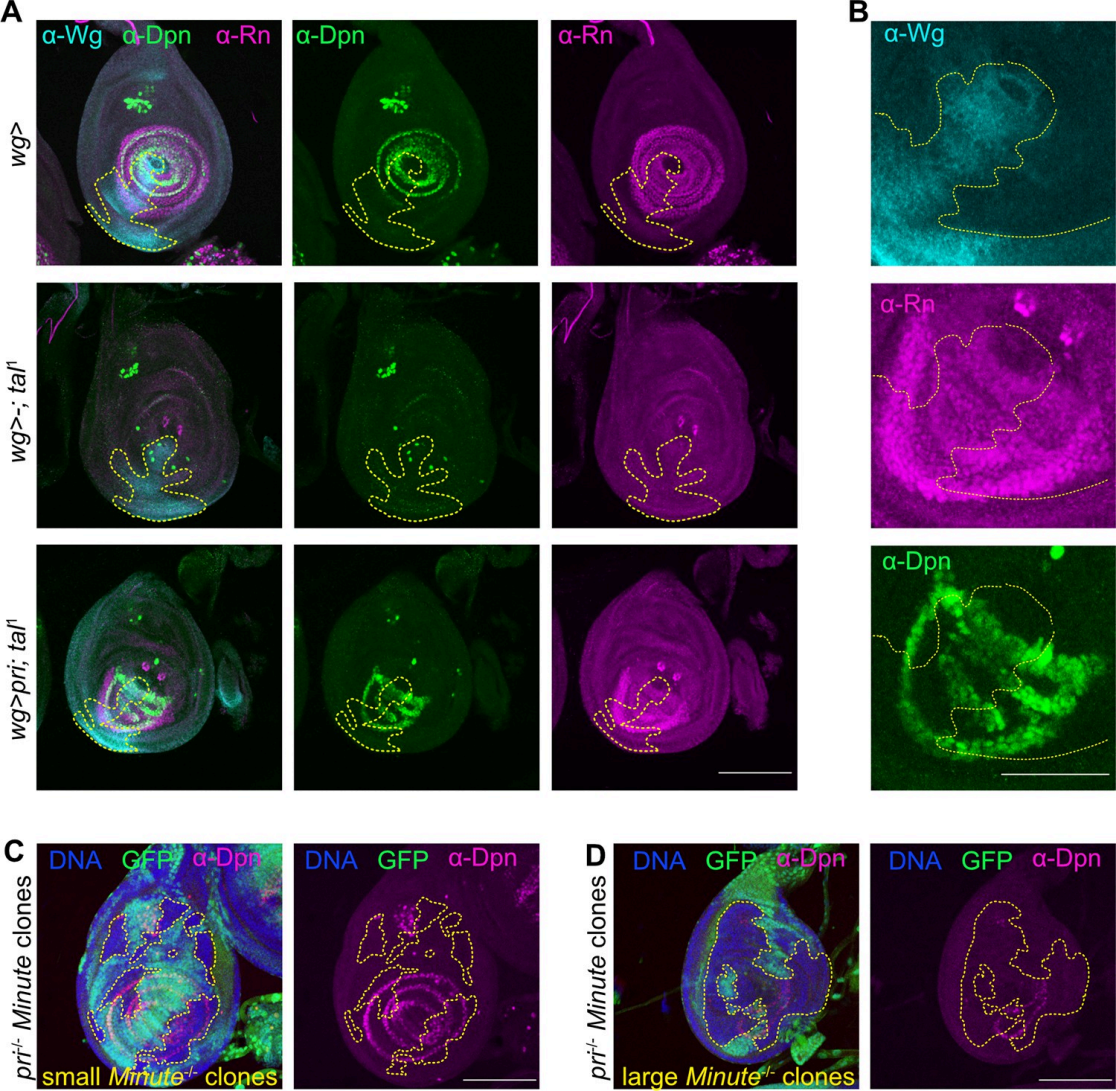
Cell non-autonomous properties of Pri peptides synchronize transcriptional program and signaling pathways in tarsus. (A) Rescue experiments have been conducted by expressing ectopically *pri* in *tal*^1^ mutant background under the *wingless*-Gal4 driver (*wg*). Wg staining delimits the region where *pri* is expressed (outlined by the yellow dashed-line). Rn and Dpn are used as read-out of tarsal patterning and Notch signaling pathway activations. Rn and Dpn are normally patterned in the control disc, whereas they are absent in the *tal*^1^ mutant. Ectopic expression of *pri* (*wg*>*pri*; *tal*^*1*^) rescues partially the *tal*^1^ phenotype since Rn and Dpn staining are restored in the *wg* region. Scale bar = 100μm. (B) Magnification on the *wg* region of the *wg*>*pri*; *tal*^*1*^ leg disc. Note that Rn and Dpn are present at the same level beyond the *wg* region, suggesting that Pri peptides induce cell non-autonomously activation of tarsal patterning. Scale bar = 50μm. (C, D) Mosaic clones for *pri*^-/-^ (*tal*^S18^) were induced in *Minute* cellular context with the expression of Flippase under the control of *Dll* driver. Generation of small clones in the tarsus does not affect Dpn patterning (C), whereas in large clones, Dpn patterning almost completely disappears (D). Scale bar = 100μm.

We conducted genetic epistasis analysis to determine the functional order of these genes in tarsal patterning. Interestingly, ectopic expression in the tarsus of *tal*^1^ mutant of activated form of EGFR or the activated EGFR ligand sSpitz [38] failed to restore Notch and EGFR signaling, or transcription factors activation (S2E Fig). Ectopic expression of *ss* is not sufficient to activate *rn*, and ectopic expression of *rn* is not sufficient to activate Notch signaling (S2F Fig). None of these actors is able to replace *pri* function, suggesting that Pri peptides are acting at several steps in this molecular cascade. Furthermore, ectopic expression of *pri* in the *wg* domain, which is expressed in a sub-region of the leg during development [28], enables Notch signaling pathway and tarsal transcriptional program activation, as visualized respectively with Dpn and Rn immunostainings (Fig 4A). Also, these factors were reactivated beyond the area of *wg* expression domain, over a distance of several cell diameters (Fig 4B), which could be due to the cell non-autonomous properties of Pri peptides, as previously observed by several laboratories [10,11,39]. Furthermore, we generated *pri*^-/-^ clones in the *Minute* cellular context and observed that clone size greatly influences the localization of Dpn and Rn. Indeed, small clones show no defect in Dpn and Rn localization, probably because Pri peptides diffuse from neighboring wild-type cells and compensate for the absence of *pri* in these small clones, whereas in large clones encompassing most of the tarsus, Rn and Dpn pattern are strongly altered (Figs 4B, 4C and S2B). This genetic approach also illustrates the non-autonomous properties of Pri peptides.

In conclusion, our data show that Pri peptides are required for the activation of signaling pathways and transcription factors that governs tarsal patterning. We propose that cell non-autonomous properties of Pri peptides could coordinate the activation of these actors within the tarsus to ensure harmonious tarsal development.

### Pri peptides controls larval disc patterning in Svb/Ubr3 independent manner

**Fig 5:**
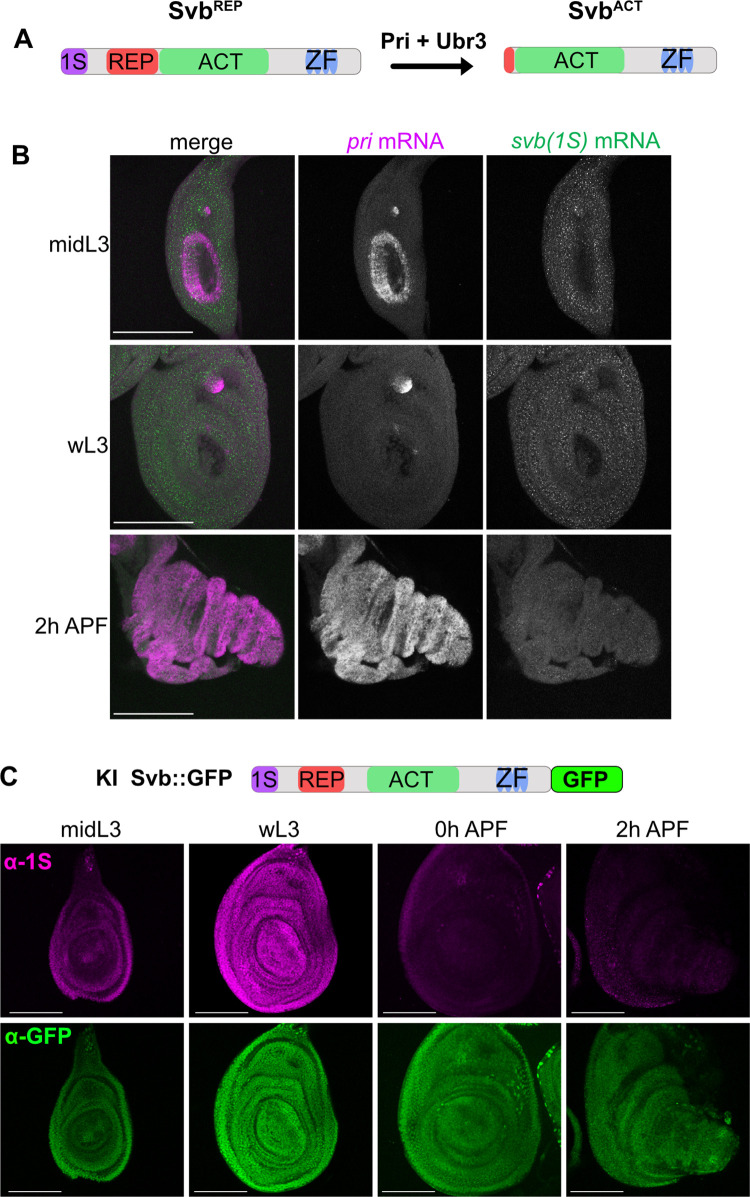
Svb is ubiquitously expressed in leg disc at larval and pupal stages. (A) Drawing representing Svb Pri/Ubr3-dependent processing. *svb* is transcribed and translated as a long repressor form (Svb^REP^), that contains the exon 1S. In embryonic epidermis, the presence of Pri peptides and Ubr3 induce the N-terminal domain degradation, leading to a shorter activator form of Svb (Svb^ACT^). (B) *svb* expression was monitored along *pri* expression by fluorescent *in situ* hybridization (smiFISH). Fluorescent *svb* probes recognize only the *1S* region of *svb*, showing that the somatic *svb* isoform is transcribed in imaginal discs. *svb* mRNA is expressed homogenously throughout the entire leg disc during larval and pupal stages and concomitantly expressed with *pri* at midL3 stage and at pupal stage. (C) Drawing representing the knock-in (KI) of GFP in the *svb* locus (*svb*::*GFP*). Anti-GFP (green) and anti-1S (purple) immunostainings on *svb*::*GFP* KI at larval stages (midL3 and wL3) and at pupal stages (0h and 2hAPF) show that Svb is ubiquitously localized in the leg disc and is in the Svb^REP^ form at larval stage and in its processed form Svb^ACT^ at the pupal stage, as confirmed by the absence of 1S signal. Scale bar = 100μm.

To go further, we then investigated whether the roles of Pri peptides in leg development during larval stage were dependent on Svb and Ubr3, the partners identified for mediating Pri functions for trichome formation during epidermal differentiation [15,16] (Fig 5A). Both *Ubr3* and *svb* are expressed during larval and pupal stages at comparable levels (S3A Fig). The *svb* mRNA and *pri* mRNA patterns during larval stage were visualized by fluorescent *in situ* hybridization (Fig 5B). While *pri* mRNA expression is transient at midL3 stage in the presumptive tarsal region and continuous throughout the disc from the onset of the pupal stage, *svb* mRNA is ubiquitously expressed at the same level throughout the leg disc during larval and 2h APF stages (Fig 5B). To go further and analyze the endogenous pattern of Svb protein in the leg disc, we generated a fly line in which the endogenous Svb was tagged to the GFP at the C-terminal position (KI *svb*::*GFP*, Fig 5C). Immunostainings against both GFP protein and 1S domain, which is specific from the Svb^REP^ [15], allow to visualize Svb^REP^ form (1S and GFP positive) and Svb^ACT^ form (only GFP positive) during leg disc development. We observed it is present as the repressor form in the larval stage and as the activator form in the pupal stage (Figs 5C, S3B and S3C). Strikingly, Svb, which is under its full-length repressive form at larval stage, is fully degraded at the timing of transient *pri* expression (S4A Fig). This complete degradation is dependent of *pri* because in *tal*^1^ mutant, Svb protein persists (S4A Fig). To test whether Svb disappearance has a role in tarsal patterning, we ectopically expressed either Svb^REP^, the full length form of Svb with 3 lysines mutated to inhibit its Pri-dependent processing [16], or the Svb^ACT^, whose amino acid sequence corresponds exactly to the Pri-dependent processed form [15]. Their expression in larval leg disc has no detectable effect on Rn pattern (S4B Fig). In addition, expression of Svb^REP^ or Svb^ACT^ does not modify the Dpn pattern (S5B Fig), on the contrary of germinal isoforms of Svb, OvoA and OvoB, commonly previously used to mimic repressor and activator forms of Svb in somatic tissues (S5A and S5C Fig). These experiments reveal that Svb^REP^ and Svb^ACT^ are better suited to faithfully reproducing the effects of repressor and activator forms of Svb in somatic tissues. Therefore, these results suggest that the transient absence of Svb at midL3 appears to not have a role in tarsal patterning.

**Fig 6:**
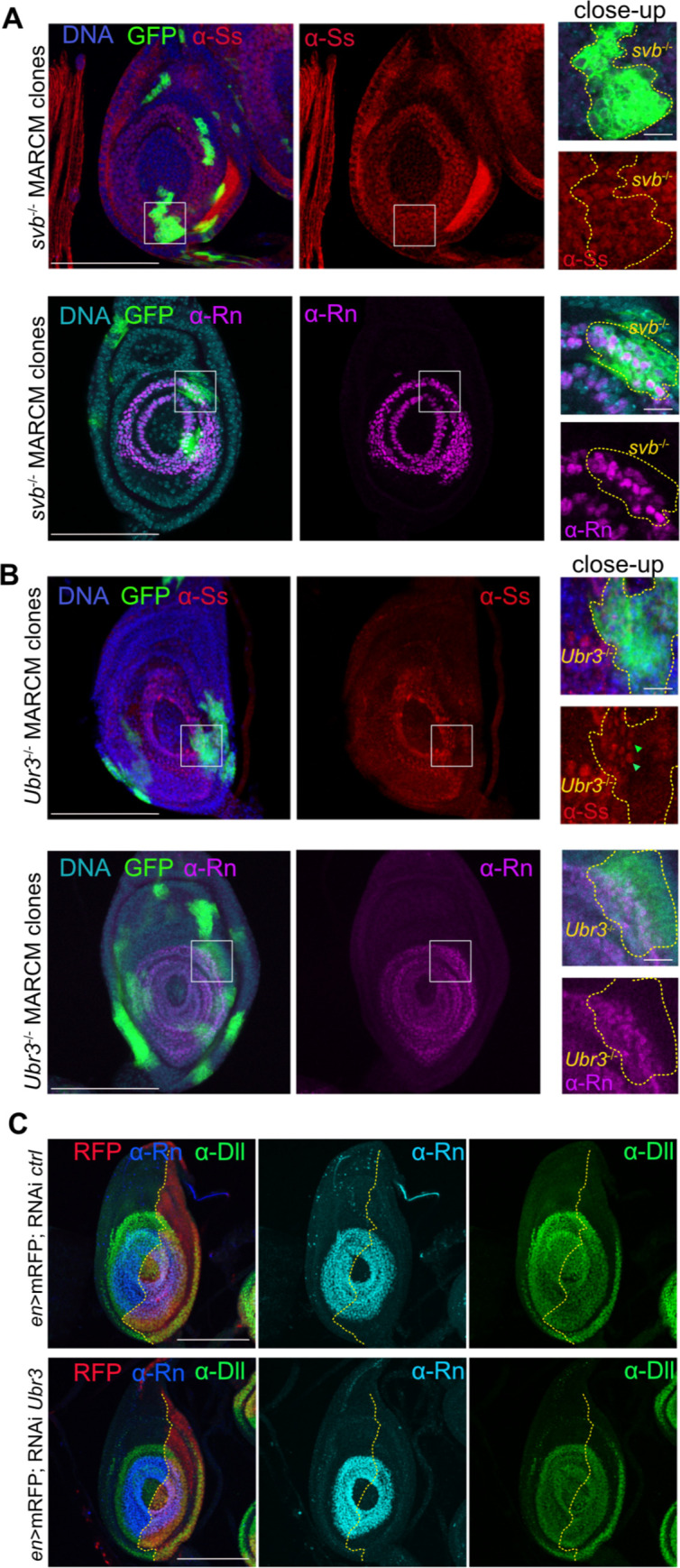
Pri peptides activate tarsal TF and EGFR and Notch signaling pathways in Svb/Ubr3 independent manner. (A) Ss and Rn were stained in *svb*^-/-^ (*svb*^PL107^) MARCM clones in L3 leg discs. Clones are in green and outlined in yellow in the close-up. In *svb*^-/-^ clones, both Ss and Rn proteins remain. (B) Ss and Rn were stained in *Ubr3*^-/-^ (*Ubr3*^B^) MARCM clones in L3 leg discs. In the *Ubr3*^-/-^ clones, both Ss and Rn proteins remain, though the level of Ss is lower (green arrow-heads). (A,B) White square highlights the region displayed in the close-up showed on the right of the panel. Scale bar = 100μm, scale in the close-up = 10μm. (C) UAS-RNAi *luciferase* (*ctrl*) or UAS-RNAi *Ubr3* were expressed under the control of *Engrailed*-Gal4 driver visualized in red (*en*>mRFP). The yellow line delimits the anterior and the posterior regions. Rn pattern remains unchanged when *Ubr3* is depleted. Scale bar = 100μm.

As Svb is ubiquitously localized in the leg disc at larval stage the majority of the time, we generated *svb* loss of function by generating mutant clones or inducing RNAi and stained leg discs to analyze the effects on tarsal patterning and Notch signaling. Persistence of Ss and Rn TFs or Dpn proteins in the absence of *svb* demonstrate that Svb is not required for their activation (Figs 6A, S6A, S6B and S6D).

We then examined the role of Ubr3 in tarsal patterning using the same genetic approaches, either by generating *Ubr3* mutant clones or by inducing loss of function by RNAi. Notch pathway is not affected by loss of *Ubr3*, as visualized by the persistence of Dpn in RNAi- depleted region or in mutant clones (S6A and S6C Fig). Also, depletion of *Ubr3* by RNAi does not modify Rn pattern (Fig 6C). Since the absence of *Ubr3* leads to apoptosis [40], the slight variation on Rn protein levels might be the consequence of a deleterious cellular context. Additionally, Ss and Rn are still present in the *Ubr3*^-/-^ clones (Figs 6B and S6E), thus supporting that Ubr3 is not required for mediating *pri* function in the larval leg disc.

Our results reveal that Pri peptides functions in patterning during the larval stage are not mediated by Svb and are independent of Ubr3, revealing the existence of additional Pri molecular targets.

### Pri peptides induce Svb processing in Ubr3 dependent manner at pupal stage

**Fig 7:**
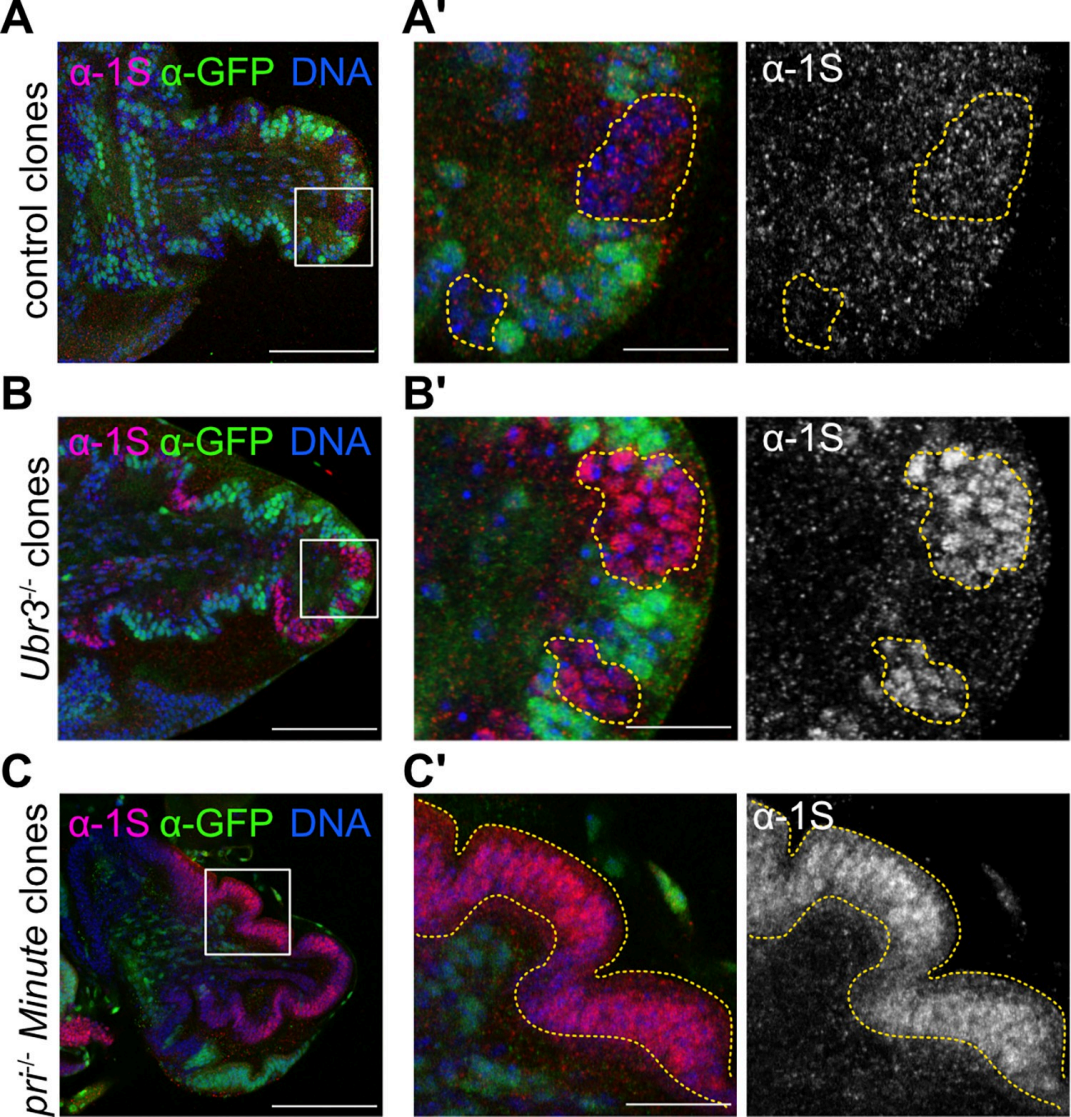
Pri and Ubr3 are required for Svb processing at the larval-pupal transition. Anti-1S immunostaining in the pupal leg disc where mosaic clones for *pri*^-/-^ (*tal*^S18^) and *Ubr3*^/-^ (*Ubr3*^B^) were induced. The clones are indicated by the absence of GFP and contoured by the yellow dashed-line. The DNA is marked in blue, 1S in red and the GFP in green, white square highlights the region displayed in the close-up (A’-C’). (A, A’) In control clones, Svb is processed as 1S signal is lost. (B, B’) In *Ubr3*^-/-^ clones, 1S staining is remaining, showing that Svb^REP^ is not processed. (C, C’) p*ri*^-/-^ mutant clones have been generated in *Minute* background with the Flippase under the control of *Dll*^EM212^-Gal4 in order to get large clones (if *pri*^-/-^ clones are too small, they are behaving like control clones). The absence of GFP indicates that almost all the leg is clonal (yellow dashed-line) and positive for anti-1S signal. (A- C) Scale bar = 100μm. (A’- C’) scale bar = 20μm.

We observed that Svb full length form persists during larval stage and is processed at larval-pupal transition at the time of tarsus eversion (Fig 5C). Furthermore, we ectopically induced *svb* depletion by RNAi in the posterior part of the leg and we observed that Svb is processed homogenously in the pupal epithelium of the leg and that there is no persistence of the full-length repressor form (S3C Fig). As Pri peptides and Ubr3 induce Svb processing in epidermis, switching the Svb^REP^ to the Svb^ACT^ forms [16], we tested whether Svb processing at larval-pupal transition was dependent of Pri and Ubr3. We generated *Ubr3*^-/-^ clones in the leg disc and showed that Svb processing relies on Ubr3 as shown by the persistence of the 1S signal at pupal stage (Fig 7A, 7A’, 7B and 7B’). To test the role of *pri*, we generated *pri*^-/-^ clones in the *Minute* cellular context to obtain large clones and avoid rescue of mutant cells by neighboring wild-type cells, due to the cell non-autonomous property of the Pri peptides. We observed also the persistence of the 1S signal in *pri*^-/-^ mutant clones (Fig 7C and 7C’). Therefore, Pri peptides are required at pupal stage to induce Ubr3-dependent Svb processing. The function of Pri/Ubr3/Svb module is reiterated during development, specifically in the imaginal leg discs during the larval-pupal transition when *pri* expression is strongly reactivated.

### Pri peptides control cell survival during pupal stages

**Fig 8:**
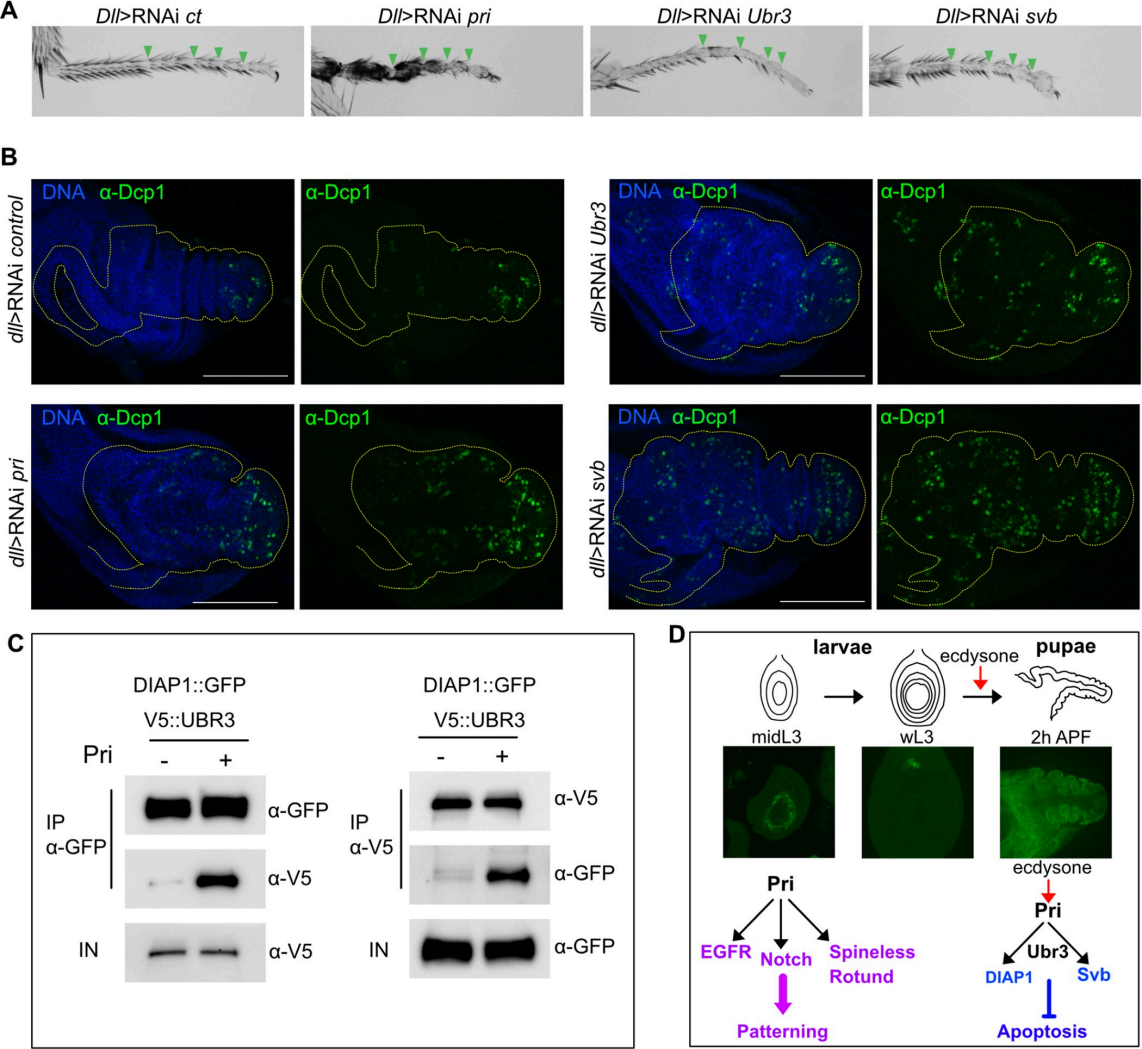
Pri peptides, Svb and Ubr3 protect cells from apoptosis at pupal stage. (A) Depletion of *pri*, *Ubr3 or svb* in the tarsus is induced by RNAi specifically at pupal stage under the control of *Dll*^EM212^-Gal4 driver and *tub*-Gal80^ts^ when larvae at wL3 stage are shifted to the restrictive temperature (29°C). Depletion of these genes alters leg morphogenesis to a different extend. Loss of *pri* induces abnormal joint formation (marked with green arrowheads), cuticle defects and loss of tissue integrity, which resembles to necrosis. Loss of function of *Ubr3* leads to growth and cuticle defects, and abnormal joints. In the absence of *svb*, the segments of the tarsus are shorter, the trichome pattern is affected and the joint are not properly formed. (B) Depletion of *pri*, *Ubr3* and *svb* were induced by RNAi at wL3 stage under the the control of *Dll*^EM212^-Gal4 driver and *tub*-Gal80^ts^. The *Dll* domain, stained by the presence of UAS-GFP, is outlined with the yellow dashed-line. Anti-Dcp-1 antibody stains apoptotic cells (in green) in pupal leg disc, that are present in the control leg disc (RNAi *luciferase*), specifically in the pretarsus, as often described. Depletion of *pri*, *Ubr3* or *svb* induces an increase in the number of apoptotic cells. Scale bar = 100μm. (C) Co-immunoprecipitation (IP) of Ubr3::V5 and DIAP1::GFP with or without Pri peptides. DIAP1::GFP and Ubr3::V5 are co-expressed in S2 cells with or without *pri*, and then co-immunoprecipitated with anti-GFP (left panel) or anti-V5 (right panel) antibodies. In the absence of *pri*, DIAP1 and Ubr3 co-interact barely. With Pri peptides, this interaction strongly increases. (D) Model of Pri peptide functions during leg development: *pri* expression is spatio-temporally regulated during leg disc development. During L3 stage, Pri peptides activate Rotund and Spineless transcription factors, and EGFR and Notch signaling pathways. Therefore, Pri peptides coordinate transcriptional program and signaling pathways to ensure tarsal patterning. Then at larval-pupal transition, when *pri* is reactivated in the leg disc by ecdysone signaling, it induces Svb processing in a Ubr3 dependent manner. *Pri* expression is maintained during pupal stage in the leg disc. The module Pri/Ubr3/Svb is required for cell survival, but also morphogenesis and for maintaining epithelial integrity.

Our results showed that the Pri, Ubr3 and Svb are cooperating during pupal leg development. We then depleted by RNAi each gene specifically during pupal stage using the *tub*-Gal80^ts^ system to see how their absence affects pupal leg development. Interestingly, depletion of each gene induces distinct phenotypes in terms of severity, suggesting that Pri peptides possess additional developmental functions compared to Ubr3 and Svb. Indeed, RNAi depletion of *pri* leads to a more severe phenotype than the depletion of *Ubr3*, which in turn is more severe than *svb* depletion (Fig 8A). As the absence of *pri* during pupal stage induces the loss of tissue integrity, we tested whether cell death could be the cause of this phenotype. We thus stained leg disc with anti-Dcp-1, the cleaved form of the ortholog of human caspase-3, and we observed a significant increase in the number of apoptotic cells (Figs 8B and S7A), thus corroborating a role for Pri peptides in protecting cells from apoptosis. We ectopically express during pupal stage both RNAi *pri* and *miRHG*, a transgene which produces miRNAs against the pro-apoptotic genes *rpr*, *hid* and *grim* [41], to strongly inhibit the apoptosis. Despite a significant decrease in apoptosis (S7A Fig), *miRHG* rescues partially tarsus morphogenesis in the absence of *pri* (S7B Fig). Furthermore, to test if the functions of *pri* during pupal stage are mediated by Svb, we expressed Svb^ACT^ in the absence of *pri*. Interestingly, we observed that Svb^ACT^ rescues partially leg phenotype (S7B Fig), suggesting that functions of *pri* during pupal stage are partially mediated through the activation of Svb.

Then, we generated loss of function of *Ubr3* and *Svb* to test also their role in cell survival during pupal stage. As shown before [40], the absence of *Ubr3* induces increased apoptosis in pupal leg disc (Fig 8B). Also, *svb* depletion increases significantly Dcp-1 positive cells, indicating that Svb also plays a role in protecting cells from cell death (Figs 8B, S8A and S8B). We performed rescue experiments by co-expressing RNAi *svb* and the major anti-apoptotic factor DIAP1 or the viral caspase inhibitor p35 [42]. Although, expression of *DIAP1* or *p35* rescue significantly the apoptosis induced by the absence of *svb* (S8B Fig), the morphogenesis of the tarsus is partially restored (S8C Fig).

To go further, we investigated the role of the module Pri/Ubr3/Svb in the regulation of DIAP1. Since Svb was shown to protect cells from apoptosis via the regulation of *DIAP1* transcription in digestive stem cells [13,14], we performed *DIAP1* fluorescent *in situ* hybridization (smiFISH) in imaginal leg discs in the absence of *svb*. We did not observe change in the level of mRNA *DIAP-1* during the time window encompassing larval-pupal transition, suggesting an alternative mechanism by which Svb protects cells from apoptosis (S8D Fig). Interestingly, Ubr3 was shown to protect cells from apoptosis in imaginal eye disc through its interaction and protection of DIAP1 protein [40], we thus tested the influence of Pri peptides on Ubr3/DIAP1 protein interaction in *Drosophila* S2 cells. Even though weak co-immunoprecipitation between Ubr3 and DIAP1 is observed without Pri peptides, the presence of Pri increases massively the interaction (Fig 8C). Our data suggests that Pri peptides and Ubr3 are cooperating for protecting DIAP1 from degradation and prevent cells from entering in apoptosis *in vivo*.

Altogether, our data reveals that the module Pri/Ubr3/Svb is protecting the leg from cell death and is necessary for morphogenesis and preserving tissue integrity throughout the development of the pupa. Moreover, rescue experiments suggest that Pri peptides, and also Svb, have additional roles beyond protecting cell from death throughout the pupal leg development.

The ability of Pri peptides to control multiple cellular events through the activation of distinct factors within the same tissue over time illustrates their pleiotropic functions in the temporal control of development (Fig 8D).

## Discussion

We took advantage of leg development features to identify novel putative smORF peptides and carried out a functional screen. We have thus shown that the family of smORF peptides, *i*.*e*. under 100 amino acid length, represents a reservoir of novel cellular and developmental regulators. Indeed, smORF peptides have been largely overlooked in genome annotations due to their small size and have remained underinvestigated so far. Focusing on leg development, we found that the most differentially expressed gene that encodes for smORF peptides controlling leg development is the *pri*/*tal* gene. Addressing Pri smORF peptide functions during leg development allowed us to dissociate larval and pupal roles, and thus to better understand its molecular action. Through their pleiotropic functions, Pri peptides by interacting with different actors trigger distinct molecular events, which synchronize tarsal patterning and morphogenesis required for harmonious leg development over time.

### smORF peptides provide a pool of novel developmental actors

Bioinformatics analyses have identified hundreds of putative smORF peptides, which have been classified in function of their origin. We focused on genes encoding smORF peptides that have never been studied because they were recently annotated (sCDS) or classified as lncRNA or pseudogenes. Importantly, genetic tools, allowing us to deplete their function, are available for half of these genes in stock centers for the fly community, and their use allowed us to identify a significant number of potential candidates for controlling development. Interestingly, 40% of smORF peptides display a motif, that could be useful to address its biological function. More than 10% of smORF peptides appear to be addressed to mitochondria, suggesting a tendency of the smORF peptides to localize to this organelle compared to the whole proteome (6%) as previously observed [43,44]. Furthermore, the phenotypes obtained are diverse, affecting cell survival, segment fusion or tissue growth, suggesting that smORF peptides are involved in all cellular processes. Indeed, recent studies showed that they can exhibit multiple subcellular localization [45,46], with role in, for example, the regulation of calcium flux, the inhibition of protein activity [47], the antigen presentation [48] or the biogenesis of the respiratory chain [49]. Obviously, further studies are now needed to better understand the function of these smORFs. Interestingly, we found that half of these putative regulatory smORF peptides have orthologs in vertebrates (S1 Table), for instance, CG33169 (55AA) is encoding for the ortholog of human SMCO4 (59AA), a peptide of unknown function containing a transmembrane domain. Thus, *Drosophila* is a good model for identifying among the hundreds of existing smORF peptides new regulators of important cellular processes conserved in eukaryotes.

It is generally accepted that eukaryotic genes are monocistronic, *i*.*e*. they contain a single ORF. However, we found that a high proportion of predicted smORF peptides are located in the 5’UTR and 3’UTR, supporting the existence of polycistronic eukaryotic genes. Indeed, recent studies in *Drosophila* or vertebrates using mass spectrometry or ribosome profiling have shown that smORF peptides are indeed translated from the 5’ or 3’ UTR, even within the main ORF, demonstrating that polycistronic genes in eukaryotes are more widespread than expected [50–52]. This highlights the potential of RNAs to code for several proteins, giving the possibility of greatly increasing the eukaryotic proteome. The challenge now is to define criteria or experimental approaches to select, among the thousands of smORF peptides, those most likely to have important regulatory functions in development.

Finally, our approach to search for putative smORF regulatory peptides, based on bioinformatic analysis of RNAseq data, is thus handable to *Drosophila* or any other organism. The use of criteria such as differentially expressed genes, expression level, or specific physiological conditions, as in our case before and after the ecdysone peak, can restrict the analyses to a smaller pool of genes. This may also increase the chances of finding a regulatory smORF peptide with specific spatio-temporal expression, which could help identify function or potential interactors. Here, these criteria highlighted the *pri*/*tal* gene, already known to be crucial for leg development [10], thus validating our approach. As two peaks of *pri* expression occur during the development of the leg disc, we wondered whether they act with the same partners.

### Pri peptides synchronize signaling pathways and transcriptional program to ensure tarsal patterning

At midL3 stage, Pri peptides were known to control *ss* and *rn* expression [22,23]. Besides a fundamental role in initiating the tarsal transcriptional program, we found that Pri peptides are also required to activate EGFR and Notch signaling pathways. We previously identified the transcription factor Svb and the E3 ubiquitin ligase Ubr3 as the molecular targets of Pri peptides during epidermal differentiation [15,16]. Here, we showed that the Pri/Svb/Ubr3 module is not mediating functions of Pri peptides for tarsal patterning during the larval stage.

Notch activation correlates with tarsal sub regionalization and segment emergence, which occurs in a Ubr3 and Svb independent manner. Our results suggest that *pri* is required for the second wave of EGFR activation in the tarsus, not in the initial EOC in the pretarsus, by regulating directly or indirectly the expression of *rhomboid* in concentric circles. Recently, it was shown in *Drosophila* embryonic tracheae that *pri* is also required for EGFR pathway activation in dorsal branches, thus supporting our data [53]. Nevertheless, we observed that ectopic activation of the EGFR pathway is insufficient to mediate Pri functions, while EGFR seems to be required for patterning at the same time as *pri*.

Ss requires *pri* for inducing *rn* expression in the leg disc [22]. Our experiments reveal that *rn*, as well as *ss*, was not able to restore tarsal patterning in the absence of *pri* and that *rn* is required for Notch signaling, thus suggesting that Pri peptides interfere several times in the regulatory cascade. Thus, we propose that Pri peptides are required concomitantly or reiteratively to activate key players, EGFR signaling, transcription factors Ss and Rn, and Notch signaling to synchronize the molecular events governing tarsal formation. Furthermore, cell non-autonomous properties of Pri peptides may be necessary to activate Pri targets at comparable levels within presumptive tarsal domain. It seems that Pri peptides are not acting like a gradient, but rather may be like a switch that either activates or does not activate its targets.

During the larval stage, Pri peptides are acting upstream of the signaling pathways and transcriptional cascade which govern tarsal patterning. Functions of Pri peptides are not mediated by Svb and Ubr3, thus suggesting that additional Pri peptides targets, direct molecular partners and/or indirect targets may exist. We speculate that the transcription factors Dll and Sp1 could interact with Pri peptides to mediate their functions as they control the same targets [8,31,35]. However, deciphering the nature of their interaction will be a long-term effort as the interdependence of the key players and cell non-autonomous properties of Pri peptides renders difficult *in vivo* genetic approaches and interpretation of the effects of their manipulation.

### Pri/Svb/Ubr3 module ensures leg tissue integrity during pupal stage

Pri peptides molecular function in epidermal differentiation, specifically during trichome formation, is mediated by Svb transcription factor and E3 ubiquitin ligase Ubr3 [16]. Here we demonstrated that the Pri/Svb/Ubr3 module is reused during leg metamorphosis, triggered by ecdysone signaling.

As previously shown in digestive stem cells, this module is required for protecting stem cells from apoptosis [13,14]. Loss of function of one of these partners induces apoptosis during pupal stage and may be as a consequence, dramatic alteration of part of the tissue. As we showed that Pri peptides increase Ubr3/DIAP1 interaction in S2 cells, and that Ubr3 interacts with DIAP1 to protect cells from death [40], the couple Pri/Ubr3 could counteract apoptosis via promoting stabilization and protection of DIAP1 *in vivo*. However, inhibition of apoptosis or ectopic Svb^ACT^ expression at the pupal stage when *pri* is absent partially rescues leg morphogenesis, suggesting that Svb and Ubr3 mediate some of the functions of Pri peptides and that these peptides may have additional targets at the pupal stage in the leg. Even though Svb is also required for protecting cells from death, we did not find any effect of Svb on *DIAP1* expression as previously described [14]. Morphological defects induced by *svb* loss of function are partially rescued when apoptosis is inhibited, suggesting that Svb may have additional functions beyond protecting cell from death. Indeed, Svb is also required for epidermal differentiation and trichome formation not only in the tarsus, but also in the tibia [54], and for joint formation [24]. However, during the first hours of pupal leg development, we did not observe a role for Svb on Notch signaling or joint patterning as previously described [24]. It might be due to the use of different genetic tools, like the *svb*::*GFP* knock-In line, which recapitulates the endogenous pattern of Svb, and the Svb^REP^ and Svb^ACT^, which are the actual epithelial forms of Svb present in leg tissue, unlike the germline OvoA and OvoB forms used previously [24].

Interestingly, the module Pri/Ubr3/Svb is operating at different stages of development and in different tissues in *Drosophila*, for instance in embryonic epidermis and in pupal leg, in which *pri* expression is temporally regulated by the ecdysone signaling (Fig 8D) [26,39]. Moreover, the module Pri/Svb/Ubr3 is conserved among arthropods and regulates embryonic patterning [17], thus suggesting this module might be reiterated across arthropods development in different tissues and organs.

In conclusion, the plethora of molecular events regulated by Pri peptides during leg development is enabled by their pleiotropy. Indeed, they can simultaneously regulate different targets within the same tissue or even within the same cells. This pleiotropy is enhanced by their spatio-temporal transcriptional regulation, which relies on multiple enhancers [26]. For example, the pulsatile expression of *pri* in the imaginal leg disc depends on several enhancers in the larval and pupal stages, of which only the pupal enhancers are regulated by ecdysone [26]. We then propose that Pri peptides rhythm *Drosophila* development by coordinating multiple and distinct cellular processes in space and time.

## Concluding remarks

The smORF peptides now represent a set of regulatory molecules capable of controlling cellular processes involved for example in development, metabolism, immunity and pathology. Furthermore, the example of the Pri smORF peptides illustrates the ability of a single peptide to induce a plethora of effects in a spatio-temporal manner by regulating distinct actors. Currently, thousands of smORF peptides have been shown to be actively translated, revealing the incredible coding potential of our genome as a source of novel bioactive molecules.

## Material and methods

### Bioinformatic smORF peptides prediction in imaginal leg discs

Imaginal leg discs were dissected in cold PBS 1X on ice from wandering L3 larvae, before the ecdysone peak, visualized by pre-spiracle eversion, and 2 hours After Pupal Formation (APF). Total RNA was extracted with Trizol reagent (Ambion) according to the manufacturer’s protocol. Construction of RNA polyA+ bank and sequencing using paired-end 100bp reads were performed by IntegraGen. Kallisto [55] was used for pseudo alignement of reads to a reference combining the Ensembl 74 annotations and additional lincRNAs from modENCODE [56,57]. We then used Sleuth for differential expression analysis [58]. Small ORFs were predicted as in Mackowiak *et al*. [18] using the transcriptomes generated in this study. The data discussed in this article are available via the Gene Expression Omnibus (GEO) under accession number GSE225561.

### Functional screen

Loss of function were induced by crossing *Dll*^EM212-Gal4^; *tub-*Gal80^ts^ flies with lines expressing RNAi or gRNA and Cas9 under the control of UAS promoter at 29°C. Fly lines are available in Bloomington and VDRC stock centers, or were generated for this study. UAS-RNAi-*white* was used as the control. The lines giving a phenotype are listed in S1 Fig.

### Fly stocks

The *Drosophila* lines used in this study are *tal*^1^, *tal*^S18.1^, UAS-RNAi *pri* [10], *Ubr3*^B^ [16], *svb*^PL107^, UAS-OvoA, UAS-OvoB [59], *Pri*I-Gal4 (generous gift from H Chanut-Delalande), UAS-*pri* [11], *wingless*-Gal4, *dysfusion*640-LacZ [35], UAS-DIAP1, UAS-miRHG [41], UAS-p35 [42]. MARCM clones were generated by using the following fly line: *y*, *w*, *hs*-FLP, *tub*-Gal80, FRT19A; UAS-mcd8-GFP; *tub*-Gal4/TM6b [14]. The *Minute* clones in tarsus were generated by using the following line: yw; *Dll*, UAS-FLP; FRT82B, *Rps*, Ubi-GFP/Cyo-TM6b.

The following lines were available from Bloomington and VDRC stock centers: *Engrailed*-Gal4, UAS-mRFP (BL30557), UAS-RNAi *luciferase* (BL31603) and UAS-RNAi *white* (BL28980), both used as controls, UAS-RNAi *svb* (v41584), UAS-RNAi *Ubr3* (v22901, v106993).

The Knock-In of GFP protein at the C-terminal position of the Ovo/Shavenbaby protein in the endogenous locus was generated by CRISPR/Cas9 by InDroso compagny.

### Hybridisation *in situ*

SmiFISH was performed as previously described [25] and FLAP-X sequence was used to generate *pri* fluorescent probes. Probes specific from the 1S region of *svb* mRNA were synthetized by Stellaris. Larvae were dissected and fixed in PFA 4% in 25 min at room temperature, then washed in PBT (PBS 1X/0,1% Triton100X) and permeabilized 20 min in PBT (PBS 1X/0,5% Triton100X). Samples were washed in the wash buffer (4M urea in SSC 2X) and incubated with hybridization mix (4 M Urea, 8μL de SSC20X, 40μL of Dextrane 20%, 3,5 μL of Vanadyl complex at 10 mM, 1,5 μL of competitor DNA, 2,5μL of smiFISH probe and 1,5 μL of water) at 37° overnight protected from light. Samples were rinsed in the wash buffer and in SSC2X. Then, samples were washed in PBT and leg discs were dissected and mounted in vectashield medium (Vector Laboratory).

For *rhomboid in situ* hybridization, probes (sense and the anti-sense) for *rhomboid* were synthesized according to standard procedures from LD06131 plasmid. Briefly, larvae were dissected in order to keep discs in PBS1X and fixed 20 min in PFA 4% at room temperature. Samples were washed in PBT (PBS1X/0,1% TritonX100), blocked 30 min in PBT (0,3% Triton X) and washed in PBT (0,1% Triton). Samples are permeabilized in Methanol/DMSO (90%/10%). Samples are rehydrated progressively, prehybridated 1 hour in hybridization buffer (50% Formamide, 4X SSC, torula RNA 1mg/ml, Heparine 0,05μg/ml, 2% Roche Blocking Reagent, 0,1% CHAPS, 50mM EDTA, 0,1% Tween20) and incubated overnight at 65°C with the denatured probe in the hybridization buffer. Samples are washed, rehydrated progressively, incubated with anti-DIG (Roche, 1/2000). Probe is then revealed with NBT/BCIP (Promega). Leg discs were dissected and mounted in a mix PBS/Glycerol.

### Immunofluorescence

Larval and pupal imaginal discs were dissected in PBS1X and fixed in PFA 4% during 25 min at room temperature, then washed in PBS1X. Samples are blocked in PBS1X/BSA 0,3%/Triton 0,3% during 1 hour. Primary antibodies are incubated overnight at 4°C. Then, samples were washed and incubated with secondary antibodies for two hours at room temperature, then rinsed in PBS1X and mounted in Vectashield medium (Vector Laboratory).

Antibody against Rotund (Rn) was obtained by immunizing guinea-pigs with the Roe (Roughened eye) full length isoform encoded by the *rotund* gene and sharing its last 450 C-terminal residues (including five Zn fingers) with the Rn isoform [34]. Contrary to Rn, *roe* is not expressed and has no function in leg tissues [34]. GST-fused Roe was produced in E coli from a pGEX-Roe plasmid [60], purified through a glutathione column and used to immunize the guinea pigs. Crude serum was used at 1:500. The antibodies used in this study are: anti-Spineless, generously given by J Yuh Nung (1/1000); anti-Distal-less generously given by R Mann (1/500), anti-GFP (Mouse, Roche) (1/500); anti-GFP (Rabbit, Torrey Pines) (1/500); anti P-ERK (P-p44/42 MAPK, Cell Signaling Technology), (1/200); 1/50, anti-Wingless (DSHB 4D4-s), (1/50); anti-Delta (DSHB C594.9B), (1/500); anti-Dcp-1 (Cell Signaling Technology), (1/200); anti-1S, (1/1500) [16], anti-Deadpan (Abcam), (1/100), anti-βGalactosidase (Promega) 1/500. The secondary antibodies were coupled to Alexa Fluor 555, 488 or 647 (Invitrogen). The DNA was marked either with TO-PRO-3 Iodide (642/661) or DAPI (Thermofisher).

### Co-immunoprecipitation and western blotting

S2 cells were transfected with pAc-V5::Ubr3, pAc-DIAP::GFP and pMT-pri. *Pri* expression was induced by CuSO_4_ at 1mM for 2 hours. Co-immunoprecipitation and western blotting were done as previously described [16].

### Image acquisition

Experiments with fluorescent markers were obtained using microscope Leica sp8. Experiments requiring white light like *in situ* hybridization or adult leg observation are acquired with microscope Nikon Eclipse 90i.

## Supporting information

S1 Table Putative small ORF encoded peptides expressed in leg disc. Here is the list of small ORF that were bioinformatically predicted. Genomic position, name and length (in AA) are shown. Predicted motifs (signal peptide, transmembrane domain, mitochondrial targeting sequence) are also specified. The phyloCSF score is reflecting the conservation of the ORF between the 12 *Drosophila* species, and is considered to be relevant above 50. The type refers to the position of smORF. The annotated smORF, which have a name, were either functionally studied, or annotated based on the conservation of protein sequence between eucaryotes. Most CG number are smORF that were recently annotated. Pseudogene means the gene is considered as non-functional. Non-coding means that the smORF is localized in non-coding RNA. CDS means the smORF in localized within the coding sequence of a canonical gene, UTR5 upstream and UTR3 downstream of the coding sequence. Other means the smORF is localized in intron or in intergenic region. Differential expression analyses were done between RNAseq data from larval and pupal leg discs. Qval is the p-value of the statistical test, logFC is the log2fold change of expression level between the two conditions, TPM-L, transcripts per million in larval discs, TPM-P, transcripts per million in pupal discs.
(XLSX)

S1 Fig Loss of function of smORF peptides in tarsus induces multiple developmental defects. Here are shown the different phenotypes and defects obtained following depletion of smORF peptide encoding genes identified in the functional screen. Loss of function was induced by expressing UAS-RNAi, or UAS-gRNA and UAS-Cas9, under the control of *Dll*-Gal4 driver. RNAi lines used are specified on each picture with the name of the CG targeted. We observed abnormal fusion of tarsal segments, defects in tarsus growth and cuticle formation, showing that smORF peptides identified here control different cellular processes. Note that RNAi *CG43324* (BL65973) is not shown because it induces necrotic legs. Scale bar = 200μm.
(TIFF)

S2 Fig Interdependence between Pri peptides, Notch signaling and tarsal transcription factors. (A) Morphology of the tarsus of *tal*^1^ mutant is rescued when *pri* is ectopically expressed under the control of *PriI*-Gal4 driver at 18°C. (B) Rn immunostaining in *pri*^-/-^ (*tal*^S18^) clones induced in the *Minute* cellular context. Clones are indicated by the absence of GFP. The control displays no clone and Rn protein is localized in the presumptive region of the tarsus. The *pri*^-/-^ clone is large enough (outlined by the yellow dashed-line) to encompass most of the leg disc, Rn pattern is then dramatically affected. Note that Rn is activated beyond the GFP positive zone, in cells that are not expressing *pri*, showing Rn activation in cell non-autonomous manner. (C) Anti-Clawless (Clw) (1/200; [37]) staining is specific from the pretarsus and is present in *tal*^1^ mutant, showing that Pri peptides are not required for pretarsus patterning. (D) *Rotund* (*rn*) was depleted by RNAi (BL65347) specifically in the posterior region of the disc under the control of *Engrailed*-Gal4 (*En*) driver. The RNAi control (ctrl) used here is RNAi *white*. Anti-Dpn staining is absent when *rn* is deleted, showing that Rn is required for activating Notch signaling pathway. (E) Rescue experiments have been conducted by expressing ectopically in *tal*^1^ mutant background under the *pri*I-Gal4 driver, *i*.*e*. in the presumptive tarsal region at midL3 stage, either sSpitz ([38]), the secreted form of the EGFR ligand, or the activated form of EGFR lambda-top (BL 59843), to activate the EGFR pathway. We observed that Rn and Dpn remain absent. (F) Similar rescue experiments with *spineless* (*ss*) (BL78354) or *rotund* (*rn*) (BL7404) were conducted in *tal*^1^ mutant background under the *pri*I-Gal4 driver. Also, we observe that neither Ss nor Rn is sufficient to activate Notch signaling in the absence of *pri* since Dpn remains absent.
(TIFF)

S3 Fig Svb localization in imaginal leg disc. (B) RNA-seq analyses on imaginal leg discs at wandering L3 stage (wL3) and at pupal stage 2 hours APF (After Pupal Formation) show a massive up-regulation of *pri* expression, whereas *svb* and *Ubr3* expressions remain stable. (B) MARCM *svb*^-/-^ (*svb*^PL107^) clones, visualized with the GFP, were generated in L3 leg disc. Leg disc was stained with anti-1S antibody. In *svb*^-/-^ clones, outlined with yellow dashed-line, anti-1S staining disappears, showing the specificity of the anti-1S antibody. (D) Expression of RNAi *svb* in the posterior domain (*En*-Gal4) of the leg disc in KI *svb*::*GFP*, marked with the mRFP, demonstrates that endogenous Svb protein is fused with the GFP and localizes ubiquitously within the leg disc. Anti-1S staining shows that Svb is under the full length repressor form. At the larval-pupal transition, Svb is processed, and remains under the short activator form during pupal leg development.
(TIFF)

S4 Fig Svb role during larval stage. (A) Anti-1S and anti-GFP staining in KI *svb*::*GFP* and in *tal*^1^ mutant background reveal that Svb is fully degraded at midL3 in the tarsal presumptive region, marked here with the anti-Spineless (Ss) antibody. In *tal*^1^ mutant background, Svb full degradation does not occur, showing that *pri* is required in this process. (B) Svb^REP^ and Svb^ACT^ are ectopically expressed with *en*-Gal4 driver during midL3 stage to analyze the effect of Svb persistence at mid L3 stage on larval leg patterning. We do not observe change in Rn staining, suggesting that Svb disappearance at midL3 stage has no role in the activation of the tarsal transcriptional program.
(TIFF)

S5 Fig Effects of Svb somatic and germinal forms on Notch signaling. (A) Drawing representing the different isoforms transcribed by the *svb/ovo* locus. In somatic tissues, *svb* is transcribed as a long isoform with *1S* exon, which is translated into Svb^REP^ protein. In the presence of Pri peptides, this full-length protein is processed into a shorter protein, Svb^ACT^, lacking the repressor domain. In the germline, *svb*/*ovo* locus is transcribed into two shorter transcripts, *ovoA* and *ovoB*, which encode respectively for a repressor and an activator of transcription. OvoA and OvoB have been commonly used by the fly community to mimic repressor and activator forms of Svb. Note that Svb^REP^, Svb^ACT^, OvoA and OvoB differ in the length of their N-terminal domains, which may result in different biological functions. (B) Flip-out clones, visualized with the GFP and outlined with the yellow dashed-line, expressing either Svb^REP^ or Svb^ACT^ are generated in the larval leg disc. Their ectopic expression does not disturb Notch signaling, indicated by Dpn staining. (C) *OvoA* and *OvoB* are ectopically expressed in the posterior domain of the larval disc with the *en*-Gal4 driver. OvoA induces an increase in Dpn positive cells, whereas OvoB repress Dpn, thus revealing that germline isoforms greatly perturb Notch signaling, in contrast to Svb somatic forms.
(TIFF)

S6 Fig Svb and Ubr3 do not regulate Notch signaling pathway at larval stage. (A) Depletion by RNAi of *Ubr3* and *svb* specifically in the posterior region of the leg disc with the *en*-Gal4 driver does not impact Dpn patterning, both at L3 stage and 2h APF pupal stage. (B, C) *svb*^-/-^ (*svb*^PL107^) and *Ubr3*^-/-^ (*Ubr3*^B^) clones are generated in leg discs, that were stained with anti-GFP and anti-Dpn antibodies to visualize the activity of Notch signaling pathway. The clones are GFP negative. The absence of *svb* (B) or *Ubr3* (C) does not affect Notch signaling, as Dpn staining is present in clones. (D) *svb*^-/-^ (*svb*^PL107^) clones at pupal stage show also that the absence of *svb* does not affect Dpn staining. (E) *Ubr3*^-/-^ (*Ubr3*^B^) clones show that the absence of *Ubr3* does not alter Rn staining.
(TIFF)

S7 Fig Pri peptides are required for cell survival at pupal stage. (A) Graph of apoptosis quantification after depletion of *pri* in *Dll* domain, which induces increase in cell apoptosis in pupal leg disc, rescued by miRHG. The apoptotic index reflects the proportion of apoptotic cells in the *Dll* domain (visualized with UAS-GFP), stained with anti-Dcp-1, whose signal intensity is measured with ImageJ. The statistical analyze is carried out using one-way ANOVA and Prism 5 (GraphPad). RNAi *control* (*luciferase*) n = 18, RNAi *pri* n = 15, RNAi *pri*+*miRHG* n = 5. * indicates 0.05 > p ≥ 0.01, ** indicates 0.01 > p ≥ 0.001. (B) Depletion of *pri* specifically at pupal stage is performed under the control of *Dll*^EM212^-Gal4 driver and *tub*-Gal80^ts^ when larvae at wL3 stage are shifted to the restrictive temperature (29°C). The absence of *pri* induces a severe leg phenotype, characterized by a loss of joints and tissue integrity (joints are highlighted with green arrowheads). Rescue experiments with miRHG or Svb^ACT^ restore partially segment growth and cuticle formation.
(TIFF)

S8 Fig Svb is required for preventing cells to enter in apoptosis. (A) *svb* was specifically deleted in the posterior *engrailed* domain (*en*-Gal4; UAS-RNAi *svb*, outlined by the yellow dashed-line) and apoptotic cells were stained with anti-Dcp-1 antibody (in green). We observed an increase in Dcp-1 positive cells in the *engrailed* domain compared to the control domain. (B) Graph of quantification of apoptotic cells in the absence of *svb* and in rescue experiments. The apoptotic index is the ratio between the percentage of apoptotic cells present in the posterior domain of the tarsus (*engrailed*) and the percentage of apoptotic cells present in the anterior domain. Apoptotic cells are stained with anti-Dcp-1, whose signal intensity is measured with ImageJ. The statistical analyze is carried out using one-way ANOVA and Prism 5 (GraphPad). RNAi *control* (*luciferase*) n = 11, RNAi *svb* n = 21, RNAi *svb*+*DIAP1* n = 11, RNAi *svb*+*p35* n = 12, RNAi *svb*+*miRHG* n = 5. * indicates 0.05 > p ≥ 0.01, ** indicates 0.01 > p ≥ 0.001, and *** indicates p < 0.001. (C) The absence of *svb* induces shorter tarsal segments, altered joint formation and shorter trichomes (joints are highlighted with green arrowheads). Rescue experiments with *DIAP1*, and to a lesser extend with *p35*, restore segment growth and trichome length. However, segment and joint shape are partially rescued. (D) Fluorescent *in situ* hybridization in imaginal leg discs of *DIAP1* mRNA in L3 and 2 hours APF. RNAi *svb* was expressed under the control of *engrailed*-Gal4 driver (*en*>) in posterior region, visualized with mRFP (purple). No significant change in *DIAP1* mRNA level is observed. Scale bar = 100μm.
(TIFF)

S1 Data Apoptotic Index values in the absence of *pri* or *svb* and rescue experiments. (XLSX)
