# Supplementary figures and images for: The pleiotropic functions of Pri smORF peptides synchronize leg development regulators
Source: PLoS Genet. 2023 Oct 30;19(10):e1011004. doi: 10.1371/journal.pgen.1011004 (PMC10635573; doi:10.1371/journal.pgen.1011004)

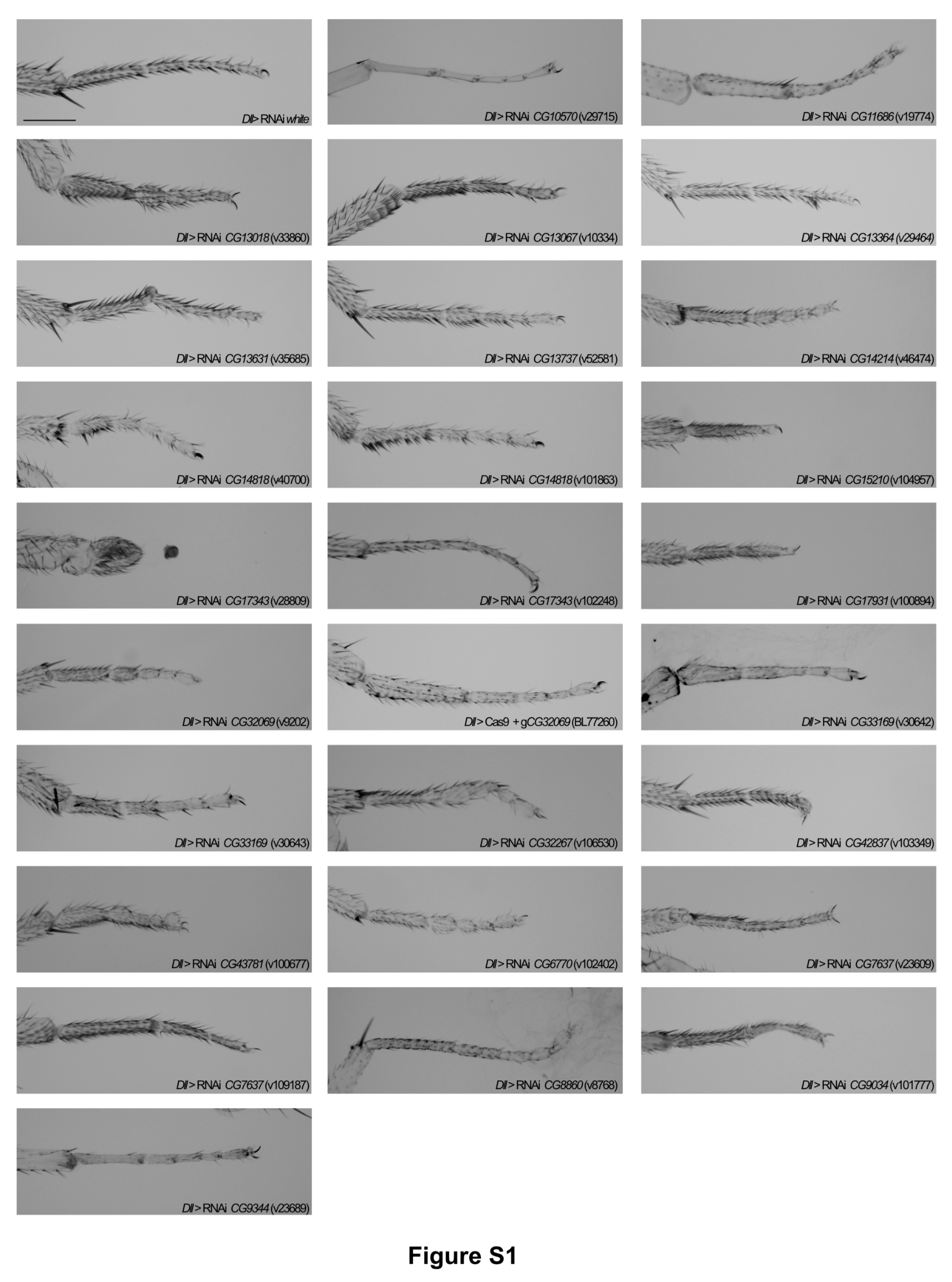

Supplement: S1 Fig — Here are shown the different phenotypes and defects obtained following depletion of smORF peptide encoding genes identified in the functional screen. Loss of function was induced by expressing UAS-RNAi, or UAS-gRNA and UAS-Cas9, under the control of Dll-Gal4 driver. RNAi lines used are specified on each picture with the name of the CG targeted. We observed abnormal fusion of tarsal segments, defects in tarsus growth and cuticle formation, showing that smORF peptides identified here control different cellular processes. Note that RNAi CG43324 (BL65973) is not shown because it induces necrotic legs. Scale bar = 200μm. (TIFF) [file pgen.1011004.s002.tiff]

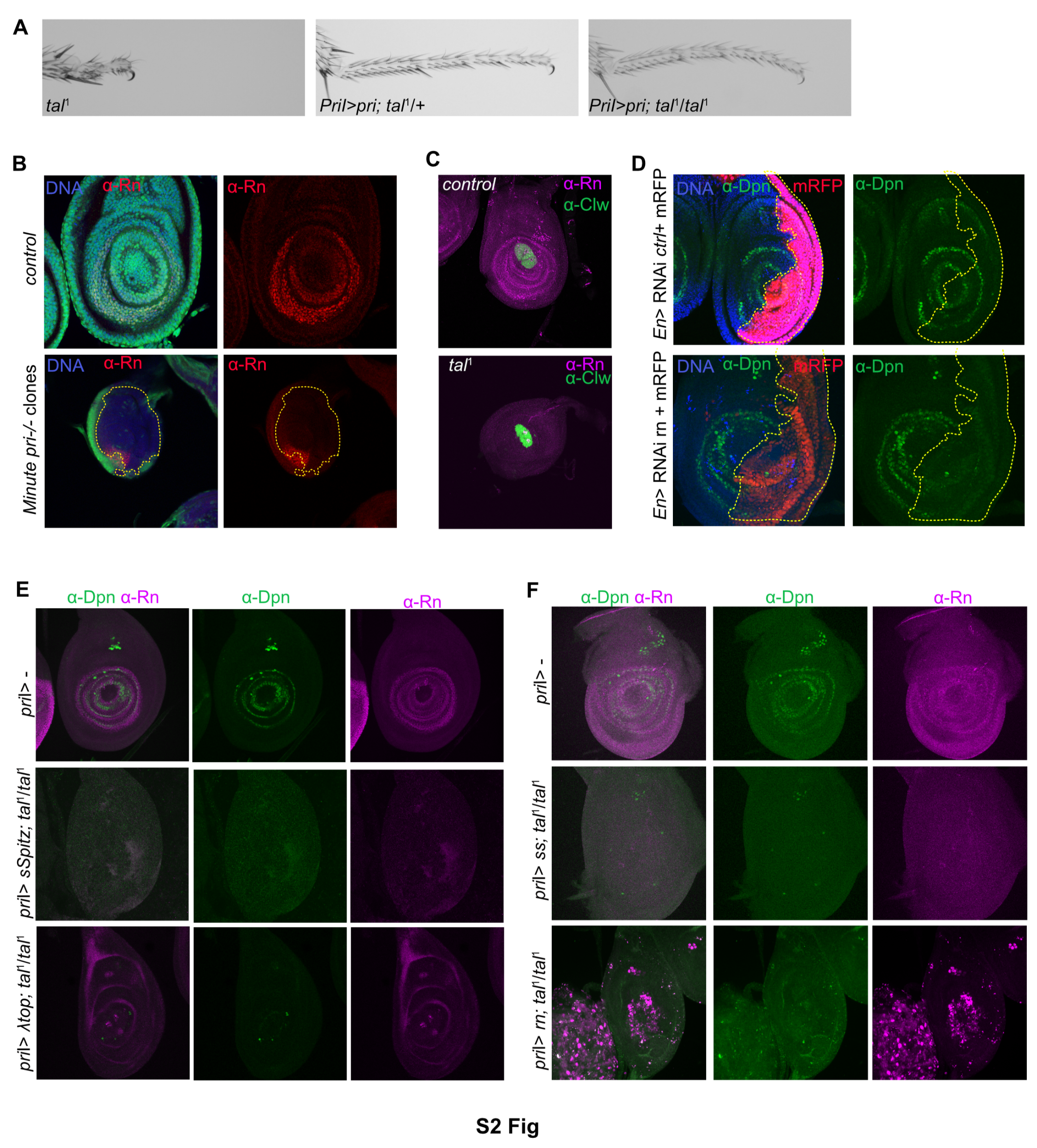

Supplement: S2 Fig — (A) Morphology of the tarsus of tal1 mutant is rescued when pri is ectopically expressed under the control of PriI-Gal4 driver at 18°C. (B) Rn immunostaining in pri-/- (talS18) clones induced in the Minute cellular context. Clones are indicated by the absence of GFP. The control displays no clone and Rn protein is localized in the presumptive region of the tarsus. The pri-/- clone is large enough (outlined by the yellow dashed-line) to encompass most of the leg disc, Rn pattern is then dramatically affected. Note that Rn is activated beyond the GFP positive zone, in cells that are not expressing pri, showing Rn activation in cell non-autonomous manner. (C) Anti-Clawless (Clw) (1/200; [37]) staining is specific from the pretarsus and is present in tal1 mutant, showing that Pri peptides are not required for pretarsus patterning. (D) Rotund (rn) was depleted by RNAi (BL65347) specifically in the posterior region of the disc under the control of Engrailed-Gal4 (En) driver. The RNAi control (ctrl) used here is RNAi white. Anti-Dpn staining is absent when rn is deleted, showing that Rn is required for activating Notch signaling pathway. (E) Rescue experiments have been conducted by expressing ectopically in tal1 mutant background under the priI-Gal4 driver, i.e. in the presumptive tarsal region at midL3 stage, either sSpitz ([38]), the secreted form of the EGFR ligand, or the activated form of EGFR lambda-top (BL 59843), to activate the EGFR pathway. We observed that Rn and Dpn remain absent. (F) Similar rescue experiments with spineless (ss) (BL78354) or rotund (rn) (BL7404) were conducted in tal1 mutant background under the priI-Gal4 driver. Also, we observe that neither Ss nor Rn is sufficient to activate Notch signaling in the absence of pri since Dpn remains absent. (TIFF) [file pgen.1011004.s003.tiff]

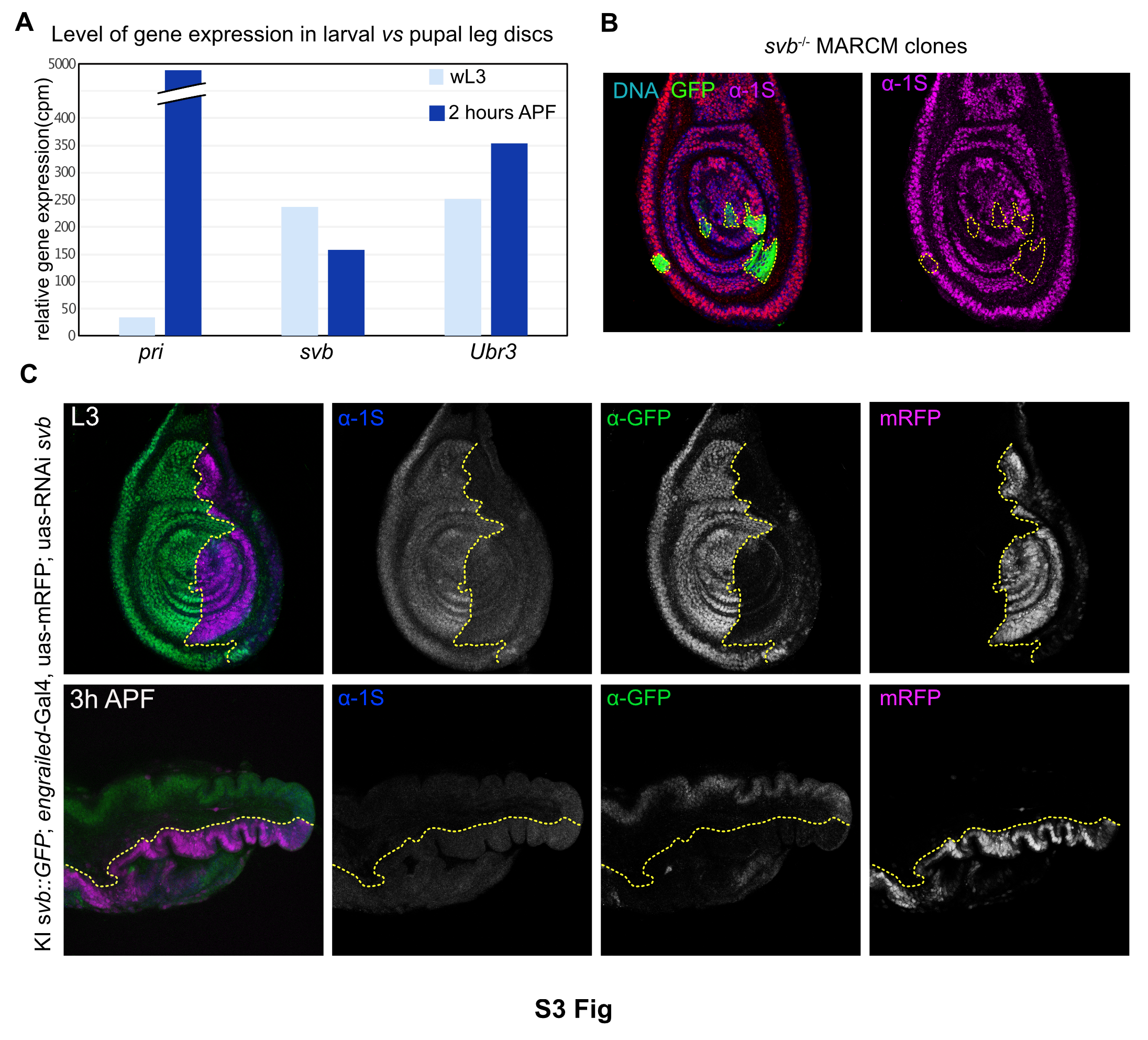

Supplement: S3 Fig — (B) RNA-seq analyses on imaginal leg discs at wandering L3 stage (wL3) and at pupal stage 2 hours APF (After Pupal Formation) show a massive up-regulation of pri expression, whereas svb and Ubr3 expressions remain stable. (B) MARCM svb-/- (svbPL107) clones, visualized with the GFP, were generated in L3 leg disc. Leg disc was stained with anti-1S antibody. In svb-/- clones, outlined with yellow dashed-line, anti-1S staining disappears, showing the specificity of the anti-1S antibody. (D) Expression of RNAi svb in the posterior domain (En-Gal4) of the leg disc in KI svb::GFP, marked with the mRFP, demonstrates that endogenous Svb protein is fused with the GFP and localizes ubiquitously within the leg disc. Anti-1S staining shows that Svb is under the full length repressor form. At the larval-pupal transition, Svb is processed, and remains under the short activator form during pupal leg development. (TIFF) [file pgen.1011004.s004.tiff]

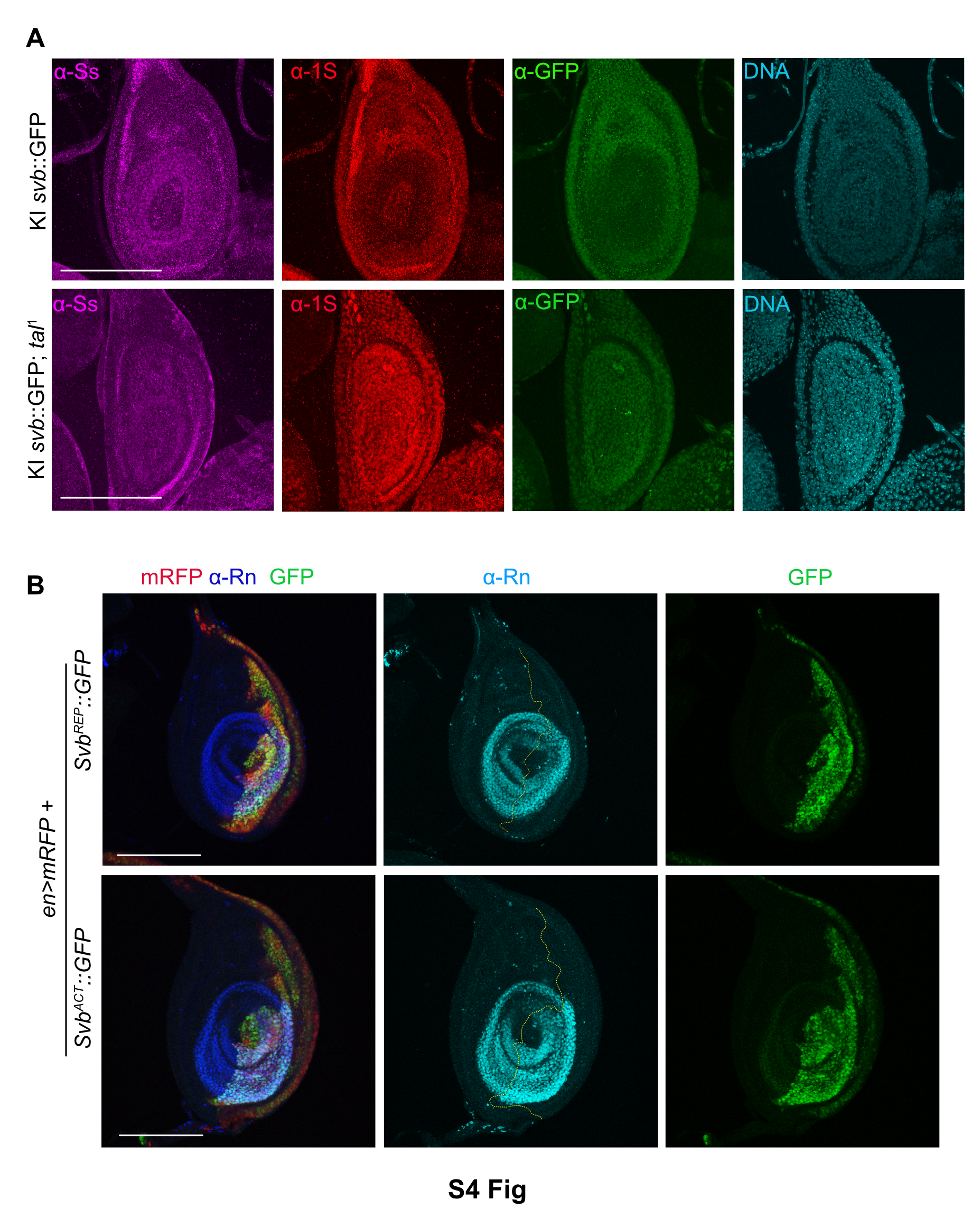

Supplement: S4 Fig — (A) Anti-1S and anti-GFP staining in KI svb::GFP and in tal1 mutant background reveal that Svb is fully degraded at midL3 in the tarsal presumptive region, marked here with the anti-Spineless (Ss) antibody. In tal1 mutant background, Svb full degradation does not occur, showing that pri is required in this process. (B) SvbREP and SvbACT are ectopically expressed with en-Gal4 driver during midL3 stage to analyze the effect of Svb persistence at mid L3 stage on larval leg patterning. We do not observe change in Rn staining, suggesting that Svb disappearance at midL3 stage has no role in the activation of the tarsal transcriptional program. (TIFF) [file pgen.1011004.s005.tiff]

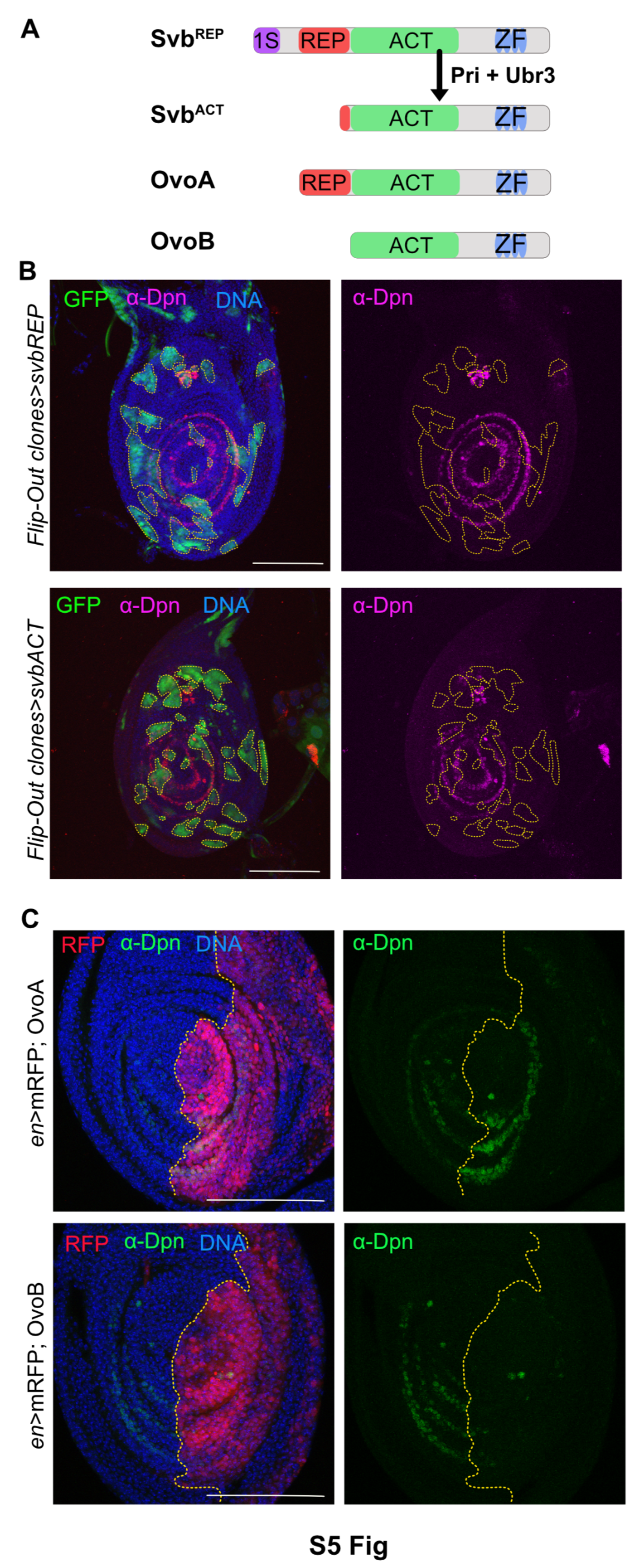

Supplement: S5 Fig — (A) Drawing representing the different isoforms transcribed by the svb/ovo locus. In somatic tissues, svb is transcribed as a long isoform with 1S exon, which is translated into SvbREP protein. In the presence of Pri peptides, this full-length protein is processed into a shorter protein, SvbACT, lacking the repressor domain. In the germline, svb/ovo locus is transcribed into two shorter transcripts, ovoA and ovoB, which encode respectively for a repressor and an activator of transcription. OvoA and OvoB have been commonly used by the fly community to mimic repressor and activator forms of Svb. Note that SvbREP, SvbACT, OvoA and OvoB differ in the length of their N-terminal domains, which may result in different biological functions. (B) Flip-out clones, visualized with the GFP and outlined with the yellow dashed-line, expressing either SvbREP or SvbACT are generated in the larval leg disc. Their ectopic expression does not disturb Notch signaling, indicated by Dpn staining. (C) OvoA and OvoB are ectopically expressed in the posterior domain of the larval disc with the en-Gal4 driver. OvoA induces an increase in Dpn positive cells, whereas OvoB repress Dpn, thus revealing that germline isoforms greatly perturb Notch signaling, in contrast to Svb somatic forms. (TIFF) [file pgen.1011004.s006.tiff]

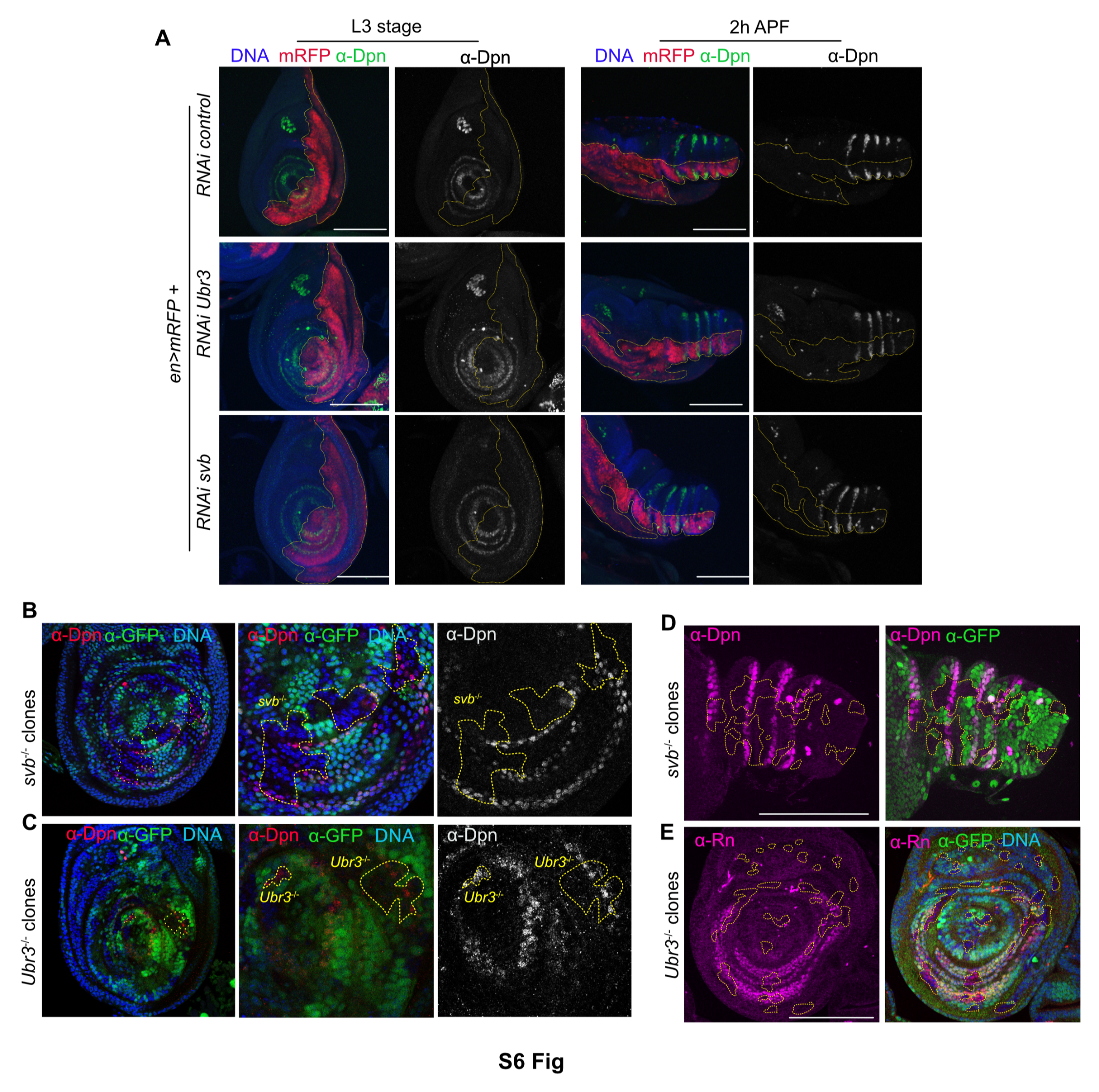

Supplement: S6 Fig — (A) Depletion by RNAi of Ubr3 and svb specifically in the posterior region of the leg disc with the en-Gal4 driver does not impact Dpn patterning, both at L3 stage and 2h APF pupal stage. (B, C) svb-/- (svbPL107) and Ubr3-/- (Ubr3B) clones are generated in leg discs, that were stained with anti-GFP and anti-Dpn antibodies to visualize the activity of Notch signaling pathway. The clones are GFP negative. The absence of svb (B) or Ubr3 (C) does not affect Notch signaling, as Dpn staining is present in clones. (D) svb-/- (svbPL107) clones at pupal stage show also that the absence of svb does not affect Dpn staining. (E) Ubr3-/- (Ubr3B) clones show that the absence of Ubr3 does not alter Rn staining. (TIFF) [file pgen.1011004.s007.tiff]

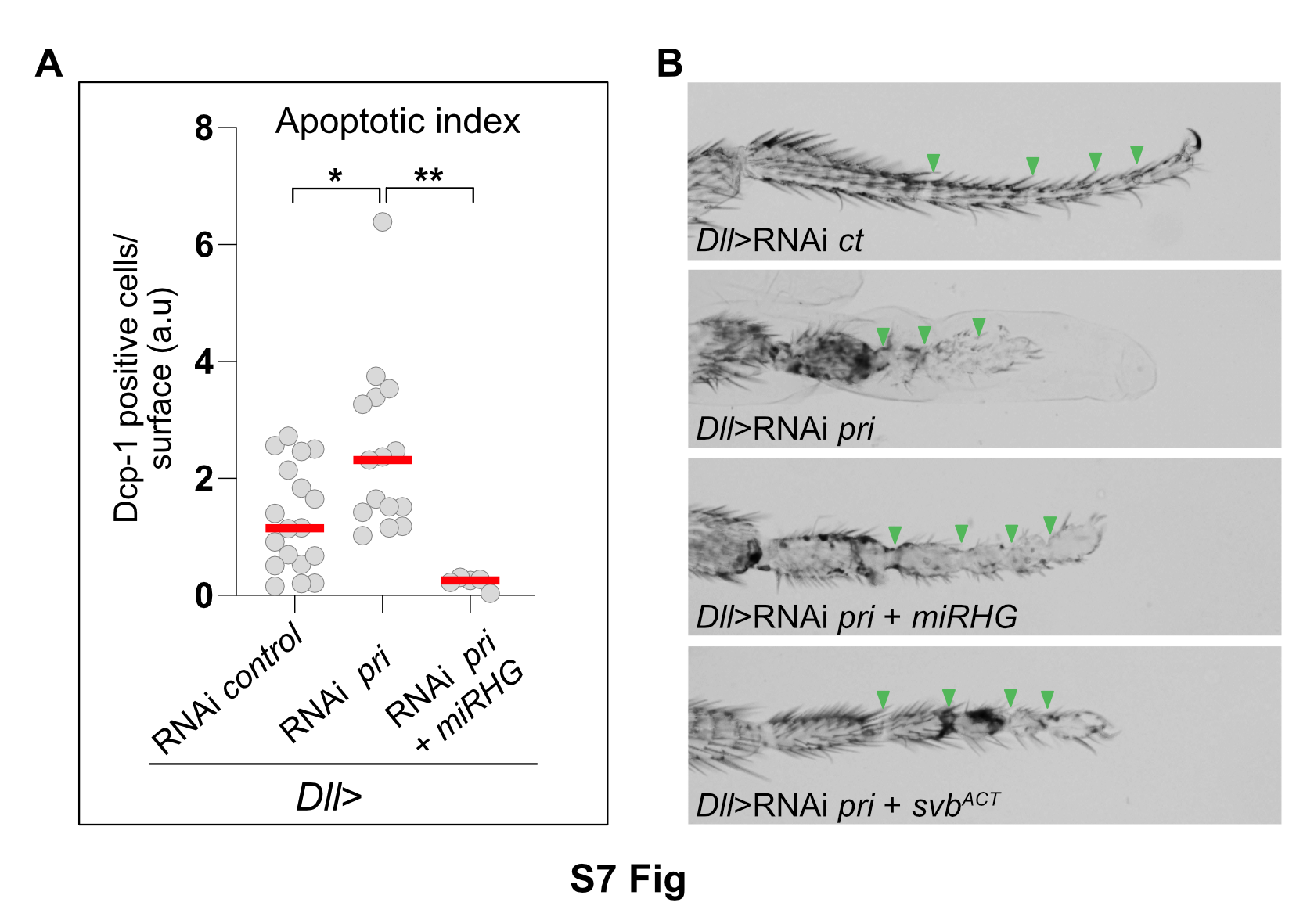

Supplement: S7 Fig — (A) Graph of apoptosis quantification after depletion of pri in Dll domain, which induces increase in cell apoptosis in pupal leg disc, rescued by miRHG. The apoptotic index reflects the proportion of apoptotic cells in the Dll domain (visualized with UAS-GFP), stained with anti-Dcp-1, whose signal intensity is measured with ImageJ. The statistical analyze is carried out using one-way ANOVA and Prism 5 (GraphPad). RNAi control (luciferase) n = 18, RNAi pri n = 15, RNAi pri+miRHG n = 5. * indicates 0.05 > p ≥ 0.01, ** indicates 0.01 > p ≥ 0.001. (B) Depletion of pri specifically at pupal stage is performed under the control of DllEM212-Gal4 driver and tub-Gal80ts when larvae at wL3 stage are shifted to the restrictive temperature (29°C). The absence of pri induces a severe leg phenotype, characterized by a loss of joints and tissue integrity (joints are highlighted with green arrowheads). Rescue experiments with miRHG or SvbACT restore partially segment growth and cuticle formation. (TIFF) [file pgen.1011004.s008.tiff]

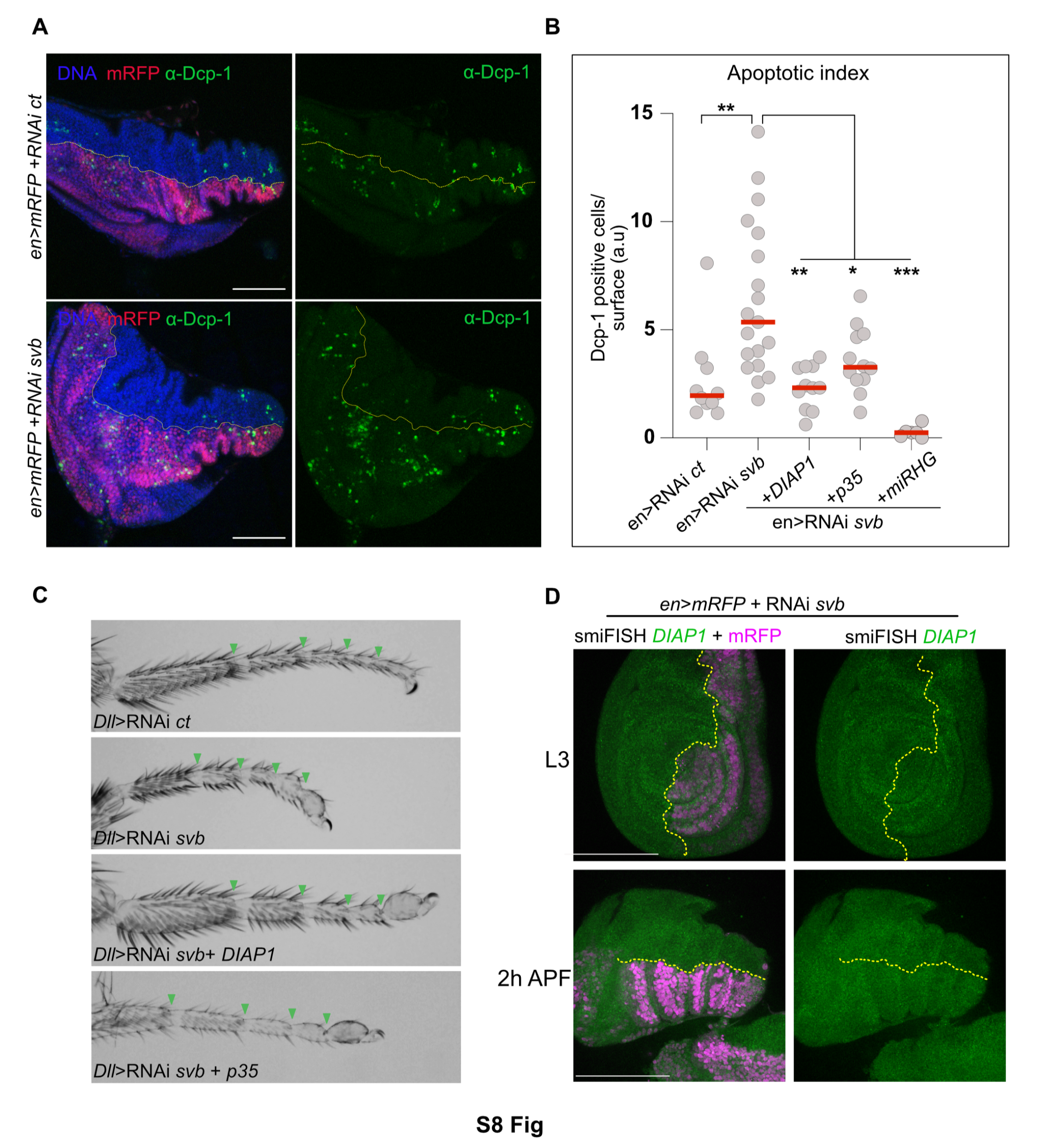

Supplement: S8 Fig — (A) svb was specifically deleted in the posterior engrailed domain (en-Gal4; UAS-RNAi svb, outlined by the yellow dashed-line) and apoptotic cells were stained with anti-Dcp-1 antibody (in green). We observed an increase in Dcp-1 positive cells in the engrailed domain compared to the control domain. (B) Graph of quantification of apoptotic cells in the absence of svb and in rescue experiments. The apoptotic index is the ratio between the percentage of apoptotic cells present in the posterior domain of the tarsus (engrailed) and the percentage of apoptotic cells present in the anterior domain. Apoptotic cells are stained with anti-Dcp-1, whose signal intensity is measured with ImageJ. The statistical analyze is carried out using one-way ANOVA and Prism 5 (GraphPad). RNAi control (luciferase) n = 11, RNAi svb n = 21, RNAi svb+DIAP1 n = 11, RNAi svb+p35 n = 12, RNAi svb+miRHG n = 5. * indicates 0.05 > p ≥ 0.01, ** indicates 0.01 > p ≥ 0.001, and *** indicates p < 0.001. (C) The absence of svb induces shorter tarsal segments, altered joint formation and shorter trichomes (joints are highlighted with green arrowheads). Rescue experiments with DIAP1, and to a lesser extend with p35, restore segment growth and trichome length. However, segment and joint shape are partially rescued. (D) Fluorescent in situ hybridization in imaginal leg discs of DIAP1 mRNA in L3 and 2 hours APF. RNAi svb was expressed under the control of engrailed-Gal4 driver (en>) in posterior region, visualized with mRFP (purple). No significant change in DIAP1 mRNA level is observed. Scale bar = 100μm. (TIFF) [file pgen.1011004.s009.tiff]
